# *Drosophila* NUAK functions with Starvin/BAG3 in autophagic protein turnover

**DOI:** 10.1371/journal.pgen.1008700

**Published:** 2020-04-22

**Authors:** David Brooks, Fawwaz Naeem, Marta Stetsiv, Samantha C. Goetting, Simranjot Bawa, Nicole Green, Cheryl Clark, Arash Bashirullah, Erika R. Geisbrecht

**Affiliations:** 1 Department of Biochemistry and Molecular Biophysics, Kansas State University, Manhattan, KS, United States of America; 2 Division of Pharmaceutical Sciences, University of Wisconsin-Madison, Madison, WI, United States of America; The Jackson Laboratory, UNITED STATES

## Abstract

The inability to remove protein aggregates in post-mitotic cells such as muscles or neurons is a cellular hallmark of aging cells and is a key factor in the initiation and progression of protein misfolding diseases. While protein aggregate disorders share common features, the molecular level events that culminate in abnormal protein accumulation cannot be explained by a single mechanism. Here we show that loss of the serine/threonine kinase NUAK causes cellular degeneration resulting from the incomplete clearance of protein aggregates in *Drosophila* larval muscles. In *NUAK* mutant muscles, regions that lack the myofibrillar proteins F-actin and Myosin heavy chain (MHC) instead contain damaged organelles and the accumulation of select proteins, including Filamin (Fil) and CryAB. NUAK biochemically and genetically interacts with *Drosophila* Starvin (Stv), the ortholog of mammalian Bcl-2-associated athanogene 3 (BAG3). Consistent with a known role for the co-chaperone BAG3 and the Heat shock cognate 71 kDa (HSC70)/HSPA8 ATPase in the autophagic clearance of proteins, RNA interference (RNAi) of *Drosophila* Stv, Hsc70-4, or autophagy-related 8a (Atg8a) all exhibit muscle degeneration and muscle contraction defects that phenocopy *NUAK* mutants. We further demonstrate that Fil is a target of NUAK kinase activity and abnormally accumulates upon loss of the BAG3-Hsc70-4 complex. In addition, Ubiquitin (Ub), ref(2)p/p62, and Atg8a are increased in regions of protein aggregation, consistent with a block in autophagy upon loss of NUAK. Collectively, our results establish a novel role for NUAK with the Stv-Hsc70-4 complex in the autophagic clearance of proteins that may eventually lead to treatment options for protein aggregate diseases.

## Author summary

Non-dividing muscle and nerve cells have limited options to clear harmful biological insults. One such insult is the progressive accumulation of damaged and misfolded proteins that ultimately destroy cellular function and may result in cell or organismal death. Understanding how and why normal protein turnover occurs in healthy tissue is essential for the eventual treatment of age-related and/or degenerative diseases that result from abnormal protein accumulation. Using the large, easily manipulated muscles in fruit fly larvae, we find that loss of the evolutionarily conserved NUAK protein results in cellular degeneration due to the abnormal accumulation of certain proteins, including Fil and CryAB. Moreover, we identify Stv/BAG3 and its binding partner Hsc70-4 to be crucial for NUAK function as overexpression of Stv rescues the *NUAK* degenerative muscle phenotype. This work is the first to uncover NUAK as a key regulator of protein degradation through autophagy and may provide a novel therapeutic target for the treatment of protein aggregate myopathies.

## Introduction

Proteins must fold into an intrinsic three dimensional structure to perform distinct cellular functions. Denatured or misfolded proteins can be refolded by chaperones or are subject to degradation by the ubiquitin-proteasome system (UPS) and/or the autophagosome-lysosome pathway (ALP) [1–3]. The accumulation of misfolded proteins upon genetic mutation or decreased chaperone function causes protein aggregates that are not effectively cleared by the UPS or the ALP. Environmental insults or aging may exacerbate this accumulation of misfolded proteins, resulting in disease and eventual cell death [4].

A specialized autophagy pathway, termed chaperone-assisted selective autophagy (CASA), has been verified in both *Drosophila* and mammalian systems [5–10]. The CASA complex includes BAG3 in concert with the chaperones HSC70/HSPA8 (HSP70 family), HSPB8 (small HSP family), and the ubiquitin (Ub) ligase CHIP/STUB1 [11]. CASA regulates the removal and degradation of Fil from the Z-disc in striated muscle or actin stress fibers in non-muscle cells [11–13]. The N-terminal actin-binding domain (ABD) in Fil is followed by multiple immunoglobulin (Ig)-like repeats which bind numerous proteins to link the internal cytoskeleton to the sarcolemma [14]. Tension exerted by contractile muscle tissue requires continuous folding and refolding of individual Ig-like domains in Fil, eventually damaging the ability of the protein to sense and transmit mechanical strain [11, 15]. The BAG3-HSC70 protein complex binds to the mechanosensor region (MSR) of Fil and upon detection of protein damage, CHIP ensures the addition of polyubiquitin (polyUb) moieties [12]. Rather than promoting delivery to the proteasome, these Ub chains instead recruit the autophagic Ub adapter protein p62/SQSTM1 [11]. p62 interacts with Atg8a/LC3 to induce autophagophore formation and the subsequent clearance of Fil through lysosomal degradation [16, 17]. Fil aggregates and a block in autophagosome-lysosome fusion are present in lysosomal associated membrane protein 2 (*LAMP2)*-deficient muscles, thus linking impaired autophagy to abnormal protein deposits [11, 18].

*Drosophila* NUAK encodes for a conserved serine/threonine kinase that is homologous to the mammalian kinases NUAK1/ARK5 and NUAK2/SNARK [19]. These proteins comprise a family of twelve AMP-activated protein kinase (AMPK)-related kinases (NUAK1 and 2, BRSK 1 and 2, QIK, QSK, SIK, MARK 1–4, and MELK) that share a conserved N-terminal kinase domain activated by the upstream liver kinase B1 (LKB1) [20]. NUAK1 and NUAK2 proteins are broadly expressed, but enriched in cardiac and skeletal muscle [21–24]. Muscle contraction and LKB1 phosphorylation can activate both NUAK proteins [19, 22]. NUAK2 activity is additionally stimulated by oxidative stress, AMP, and glucose deprivation in various cell types [22]. Interestingly, NUAK2 expression increases during muscle differentiation and in response to stress or in aging muscle tissue, whereas dominant-negative (DN)-NUAK2 induces atrophy [23]. Homozygous *NUAK1 KO* mice are embryonic lethal and <10% of NUAK2 homozygotes survive [25], precluding analysis of post-embryonic contributions. Because of this embryonic lethality, conditional *NUAK1 KO* mice were generated to examine muscle function [26, 27]. However, no change was observed in muscle mass or fiber size between control or muscle-specific *NUAK1 KO* mice, likely due to functional redundancy.

The presence of single NUAK orthologs in worms (Unc-82) or flies (NUAK/CG43143) allows for the study of NUAK protein function without compensation from additional family members that may mask cellular roles. Unc-82 associates with Paramyosin and likely Myosin B to promote proper myofilament assembly in *C*. *elegans* [28, 29]. The kinase domain in *Drosophila* NUAK shares 61% identity and 80% similarity to human NUAK1 and NUAK2. In flies, RNAi knockdown of NUAK phenocopies weak *Lkb1* defects in regulating cell polarity during ommatidial formation and actin cone formation in spermatogenesis [30, 31]. NUAK kinase targets or additional functions in other tissues have not been reported.

Here we identify *Drosophila* NUAK as a key regulator of autophagic protein clearance in muscle tissue. NUAK physically interacts with and phosphorylates Fil [encoded by *Drosophila cheerio (cher)]*. NUAK also genetically and biochemically interacts with the Stv-Hsc-70-4 complex and Stv overexpression is sufficient to rescue NUAK-mediated muscle deterioration. The identification of Fil as a cargo protein that abnormally accumulates in muscle tissue deficient for NUAK, Stv, Hsc70-4, and Atg8a links protein aggregation to defects in autophagic disposal.

## Results

### *NUAK* mutants (*NUAK-/-*) exhibit a degenerative muscle phenotype

**Fig 1:**
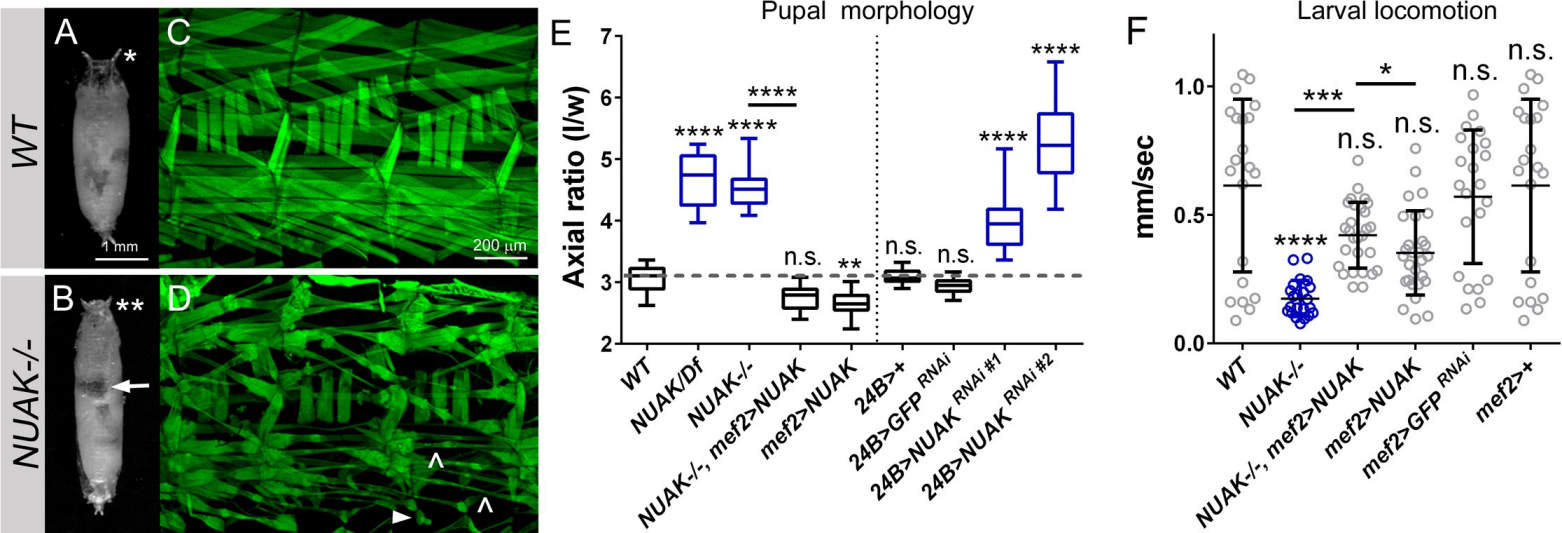
NUAK mutants are defective in muscle structure and function. (A,B) Representative pupal cases. (A) *WT* pupae exhibit a stereotypical size and shape with prominent anterior spiracles (asterisk). (B) Mutations in *NUAK* result in elongated pupae with shorter spiracles (double asterisks) and a failure in abdominal air bubble displacement (arrow). (C,D) Three hemisegments of L3 muscle pelts stained with phalloidin to visualize F-actin. (C) A regular pattern of somatic body wall muscles is apparent in *WT* fillets. (D) Drastic morphological defects, including thinner (carets) and detached (arrowhead) muscles are prevalent in *NUAK-/-*. (E) Box and whisker plot showing the axial ratios, or length (l) to width (w) proportions, of the indicated genotypes. Reductions in NUAK, either through mutations or RNAi knockdown, all exhibit a greater axial ratio compared to *WT* pupae (gray dashed line). Reintroduction of muscle-expressed NUAK rescues the elongated pupal phenotype back to *WT*. Rescue crosses were performed at 18°C (*NUAK-/-*, *mef2>NUAK* and *mef2>NUAK*), while 29°C was used to induce maximal RNAi knockdown (*24B/+*, *24B>GFP* ^*RNAi*^, *24B>NUAK* ^*RNAi*^). Statistical values directly above each genotype are compared to *WT* (left panel) or *24B* (right panel) control larvae. (F) Scatter plot depicting the locomotor ability of L3 larvae across agar plates. The motility of larvae deficient for NUAK is reduced, but can be restored with NUAK overexpression. All genotypes were reared at 18°C as rescue could be achieved at this temperature. Statistical values directly above each genotype are compared to *WT* control larvae. Mean +/- SD. (*, p<0.05; **, p<0.01; ***, p<0.005; ****p<0.001; n.s., not significant).

We and others have identified novel mutations that affect muscle structure or function using abnormal pupal morphology as a visual marker [32–35]. Contraction of body wall muscles during the larval to pupal transition results in a characteristic *WT* pupal case (**Fig 1A**), whereas the inability to contract muscles causes elongated pupae (**Fig 1B**). To identify additional regulators of muscle biology, we screened a collection of 566 EMS-induced pupal lethal mutations for abnormal pupal morphology [36]. Seven mutations in this collection exhibited an elongated and/or curved pupal phenotype. One of these mutations mapped to the previously characterized *sallamus (sls)* locus and encodes for the large muscle protein Titin, validating the functionality of our screening approach in identifying mutants that are defective in muscle structure and/or function. Deficiency (Df) mapping of the mutation in **Fig 1B**, originally designated *l(3)17289*, narrowed the region down to nine protein encoding genes within *Df(3R)BCS479* (**S1 Fig**). Sequencing of messenger RNAs (mRNA) isolated from *l(3)17289* homozygous mutants revealed a C→T transition resulting in a premature stop codon (AA829) in the uncharacterized gene *CG43143* (hereafter referred to as *NUAK*) (**S1 Fig**). Gross level examination of third larval instar (L3) fillets revealed a dramatic loss of tissue integrity in muscles homozygous for the *l(3)17289* alelle *(NUAK-/-)*, primarily characterized by thinning or detached muscles (**Fig 1C and 1D**). Targeting of two independent RNAi lines in muscle tissue (*mef2>NUAK RNAi*) each decreased NUAK transcript levels by more than 50% and showed similar defects in muscle morphology (**S2 Fig**). Together, these data strongly suggest that NUAK functions in preventing muscle tissue degeneration.

We further confirmed *NUAK* as the causative gene for defective muscle function. First we measured pupal case length/width (axial) ratios to assess muscle contraction during the larval to pupal transition. The axial ratio value of *WT* pupae was approximately 3. In contrast, mutants homozygous for the *l(3)17289* allele or this allele over the *Df(3R)BSC479* deficiency showed an axial ratio greater than 4 (**Fig 1E**). Ubiquitous (*da*-Gal4) knockdown of NUAK using two independent RNAi hairpins phenocopied these increased pupal case axial ratios (**S2 Fig**). To determine if NUAK function is muscle autonomous, we induced *NUAK RNAi* with a Gal4 driver under control of the *24B* (*held out wings*) muscle promoter and observed a failure of muscle contraction during the larval to pupal transition (**Fig 1E**). Reintroduction of full length *NUAK cDNA* into muscle tissue under control of the *mef2* promoter rescued this elongated pupal phenotype in *NUAK*-/-, confirming that NUAK is indeed the causative gene for the observed phenotypes.

In a second assay to evaluate the functional necessity of NUAK in muscle contraction, we monitored larval locomotion. *WT* L3 larvae traversed across an agar plate at an average velocity of 0.6 mm/sec (**Fig 1F**). The rate of *NUAK-/-* larvae was greatly reduced compared to *WT* or *mef2* controls, but improved upon NUAK overexpression in muscle tissue. This muscle-specific rescue of NUAK in pupal body wall contraction and larval locomotion does not rule out a role for NUAK in neuromuscular transmission. To further explore this possibility, we reduced NUAK in either muscles or neurons and assessed muscle morphology and locomotion. Muscle-specific knockdown of NUAK showed both muscle degeneration and locomotion defects (**S3 Fig**), while this same decrease in the neuronal contribution of NUAK had no effect (**S3 Fig**).

**Fig 2:**
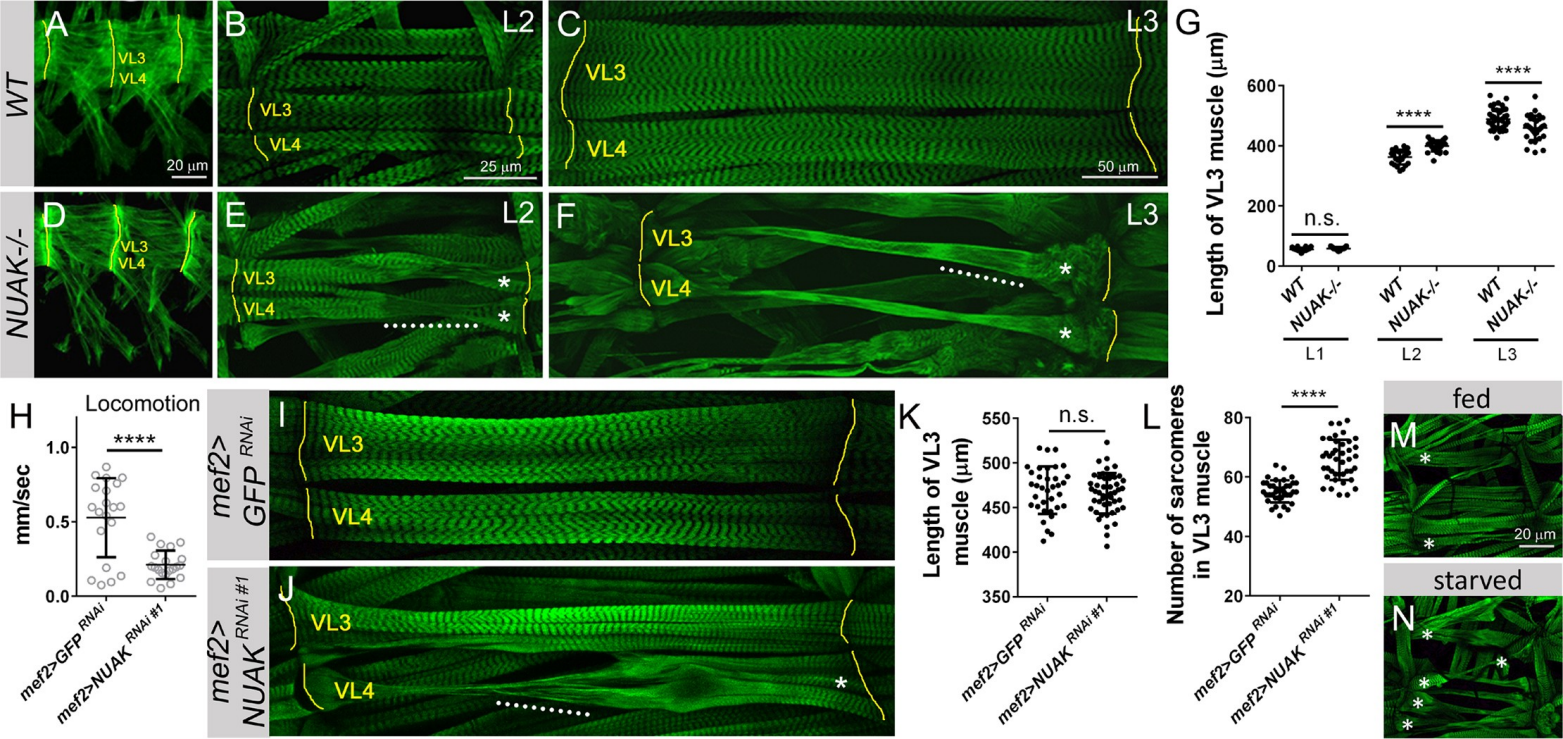
Progressive muscle degeneration is prevalent upon a loss of NUAK. (A-F) Visualization of muscles VL3 and VL4 (green) throughout embryonic and larval development. Muscle attachment sites at the hemisegment borders are denoted with yellow lines. (A,D) Two hemisegments of the embryonic musculature in stage 17 embryos are immunostained with anti-MHC. (B,C,E,F) Phalloidin-labeled *WT* or *NUAK-/-* larvae. (A) The normal muscle pattern in a *WT* embryo. (B,C) *WT* muscles are rectangular and increase in size from the L2 (B) to the L3 (C) stage. (D) The final pattern of muscles in homozygous *NUAK-/-* embryos appears *WT*. (E,F) Muscle defects, including a loss of sarcomeric patterning and thinning muscles (white dotted line) in *NUAK*-/- are apparent in L2 individuals (E) and progressively deteriorate during the L3 stage (F). Asterisks indicate affected muscles. (G) Scatter plot showing the length of individual VL3 muscles during larval development upon loss of NUAK. (H-N) Knockdown of NUAK via expression of a weaker RNAi line in muscle tissue (*mef2>UAS-NUAK* ^*RNAi #1*^). (H) Scatter plot reveals a decrease in locomotor ability upon *NUAK RNAi* compared to *GFP RNAi* controls. (I,J) Muscles VL3 and VL4 (green) are not affected in *GFP RNAi* control larvae (I), but exhibit variable defects upon *NUAK RNAi* knockdown (J, white dotted lines). Asterisks indicate affected muscles. (K,L) Scatter plots indicate that the length of VL3 muscles is not altered (K), but sarcomere number is increased, upon induction of *NUAK RNAi* (L). (M,N) One hemisegment of the larval musculature in *mef2>UAS-NUAK* ^*RNAi #1*^ larvae under fed (M) or growth-inhibited (N) conditions (n = 6 for each). The increase in abnormal phenotypes (*) when growth is stalled indicates that muscle use is the primary cause of tissue degeneration. Mean +/- SD. (****p<0.001; n.s., not significant).

Muscle morphology was next examined from the onset of myogenesis to determine the temporal progression of degeneration that resulted in the dramatic phenotypes present at the end of larval development (**Fig 1D**). While all muscle groups were affected upon loss of NUAK, we chose to follow ventral longitudinal muscles 3 (VL3) and 4 (VL4) from stage 16 in embryogenesis through the L3 stage. VL3 and VL4 are part of the innermost group of VL muscles that span each abdominal segment (**Fig 2A**) [37]. The addition and maturation of sarcomeres results in highly regular, repeated striations in second instar larval (L2) muscles (**Fig 2B**) that persist into the L3 stage (**Fig 2C**). The overall pattern of embryonic muscles appeared normal upon loss of NUAK (**Fig 2D**). Muscle abnormalities were first observed in L1 individuals (**S2 Fig**) and this cellular degeneration continued throughout the L2 (**Fig 2E**) and L3 stages (**Fig 2F**), culminating in thinner muscles devoid of typical sarcomere patterning (white dotted lines).

### NUAK-mediated muscle degeneration is independent of growth

Somatic body wall muscles undergo massive growth during larval development. To determine if NUAK-mediated muscle degeneration affects muscle size, we measured myofiber length throughout larval development. Both *WT* and *NUAK-/*- VL3 muscles were approximately the same length in the L1 stage, while NUAK-deficient muscles measured longer in L2 larvae (**Fig 2G**). This increase in muscle length was no longer apparent in *NUAK-/-* L3 muscles (**Fig 2G**), possibly due to the loss of sarcomere morphology coupled with severe tissue degeneration (**Fig 2F**). To further understand the cellular basis for differences in larval muscle length, we took advantage of the ability to modulate NUAK function using RNAi. While *NUAK* transcript levels were quantitatively similar after RNAi silencing of either *NUAK RNAi* line (**S2 Fig**), the functionally weaker *UAS-NUAK RNAi #1* insertion showed fewer degenerating muscles, allowing us to quantitate length in muscles that retained sarcomeres. First, we confirmed that induction of *NUAK RNAi* in larval muscles using the *mef2*-Gal4 driver at 25°C reduced locomotor activity (**Fig 2H**). Next, we found that the overall length of VL3 muscles were not different between *GFP RNAi* control (**Fig 2I and 2K**) or *mef2>NUAK RNAi* larvae (**Fig 2J and 2K**), demonstrating that partial loss of NUAK does not affect muscle length. However, there was an increase in sarcomere number upon reduction of NUAK (**Fig 2L**), suggesting that at least one function of NUAK may be to limit new sarcomere addition.

Alterations in nutritional status can be used to probe growth requirements during larval development. Larvae deprived of food ~ 70 hours (hrs) after egg laying (AEL) are retarded in growth, but retain the ability to crawl and may survive to adulthood, although reduced in overall body size [38]. To determine if NUAK function is linked to muscle growth or muscle use, we removed *NUAK*-/- larvae from food ~70h AEL and monitored development. While most of the *WT* larvae generated small pupae, *NUAK-/-* larvae died within 24h. To circumvent this lethality due to loss of NUAK, we performed the same experiment with weak knockdown of NUAK in muscle tissue. *NUAK RNAi* muscles showed minor defects when reared on normal food (**Fig 2M**), while the severity of these muscle phenotypes were consistently increased upon starvation (**Fig 2N**). Here we conclude that the muscle defects in *NUAK* mutants are independent of growth, but likely linked to muscle use.

### Regions devoid of myofibrillar material contain heterogeneous protein aggregates

**Fig 3:**
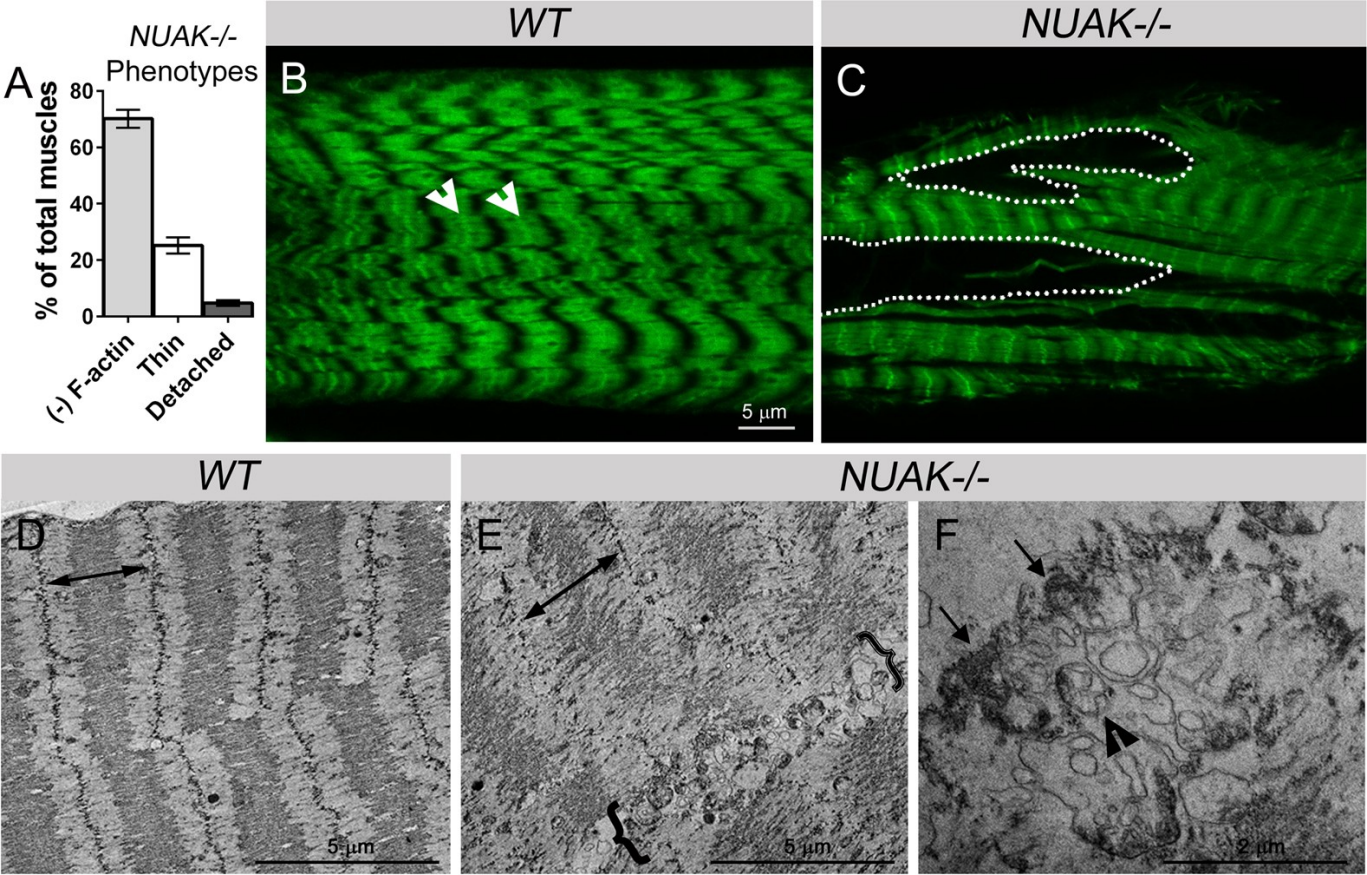
Myofibrillar material is replaced by damaged organelles and abnormal aggregates in NUAK-deficient muscles. (A) Bar graph showing the relative percentage of phenotypes present in *NUAK-/-* muscles. (B,C) High magnification images of muscle tissue stained with phalloidin (green). (B) *WT* muscle shows a stereotypical pattern of sarcomeres with prominent Z-discs (white indented arrowheads). (C) F-actin labeled myofibrillar material is absent in large regions (white dotted outlines) upon loss of NUAK. (D-F) TEM micrographs of filleted L3 muscle tissue. (D) *WT* muscles reveal organized sarcomeres with prominent Z-discs and evenly spaced sarcomeres (double arrows). (E) Wider sarcomeres (double arrows), disintegration of Z-disc structures, and the accumulation of damaged organelles (brackets) are present in *NUAK-/-* muscle. (F) High magnification micrograph reveals mitochondria with abnormal cristae (black indented arrowhead) and electron-dense protein aggregates (black arrows). Mean +/- SEM.

The severe muscle degeneration in *NUAK*-/- precluded analysis of sarcomere number (**Fig 2F**). However, these defective muscles showed a range of additional phenotypes, including thinner myofibers (white carets in **Fig 1D**; asterisks in **Fig 2E and 2F**), occasional muscle detachment (white arrowhead in **Fig 1D**), and a loss of sarcomeric patterning (dotted lines in **Fig 2E and 2F**). Thin and detached muscles represented ~25% of the defects present in *NUAK-/-* muscles (**Fig 3A**). The most prevalent phenotype upon loss of NUAK corresponded to dark regions that lacked the typical F-actin sarcomere structure. While high magnification images revealed the stereotypical repeating pattern of sarcomeres in *WT* muscles (**Fig 3B**), this same examination of *NUAK-/-* muscle tissue in areas with aberrant sarcomeric patterning (dotted lines in **Fig 2F**) failed to stain positive for F-actin structures (**Fig 3C**). This lack of phalloidin staining in *NUAK-/-* muscles could result from the complete absence of cellular material or a displacement of F-actin by other myofibrillar components. To distinguish between these two possibilities, we examined the ultrastructure of the L3 musculature using transmission electron microscopy (TEM). *WT* muscles showed evenly spaced sarcomeres with prominent Z-discs (indicated by double arrowhead in **Fig 3D**). Loss of NUAK caused disintegration of Z-disc morphology and an overall disorganization of the repeated sarcomere pattern (note extended double arrowhead in **Fig 3E**). Notably, areas within *NUAK-/-* muscles that lost sarcomere structures (brackets in **Fig 3E**) instead contained a heterogeneous mixture of damaged organelles (black indented arrowhead in **Fig 3F**) and electron-dense aggregates (black arrows in **Fig 3F**), clearly illustrating that regions devoid of myofibrillar components are replaced with the abnormal accumulation of cellular material.

**Fig 4:**
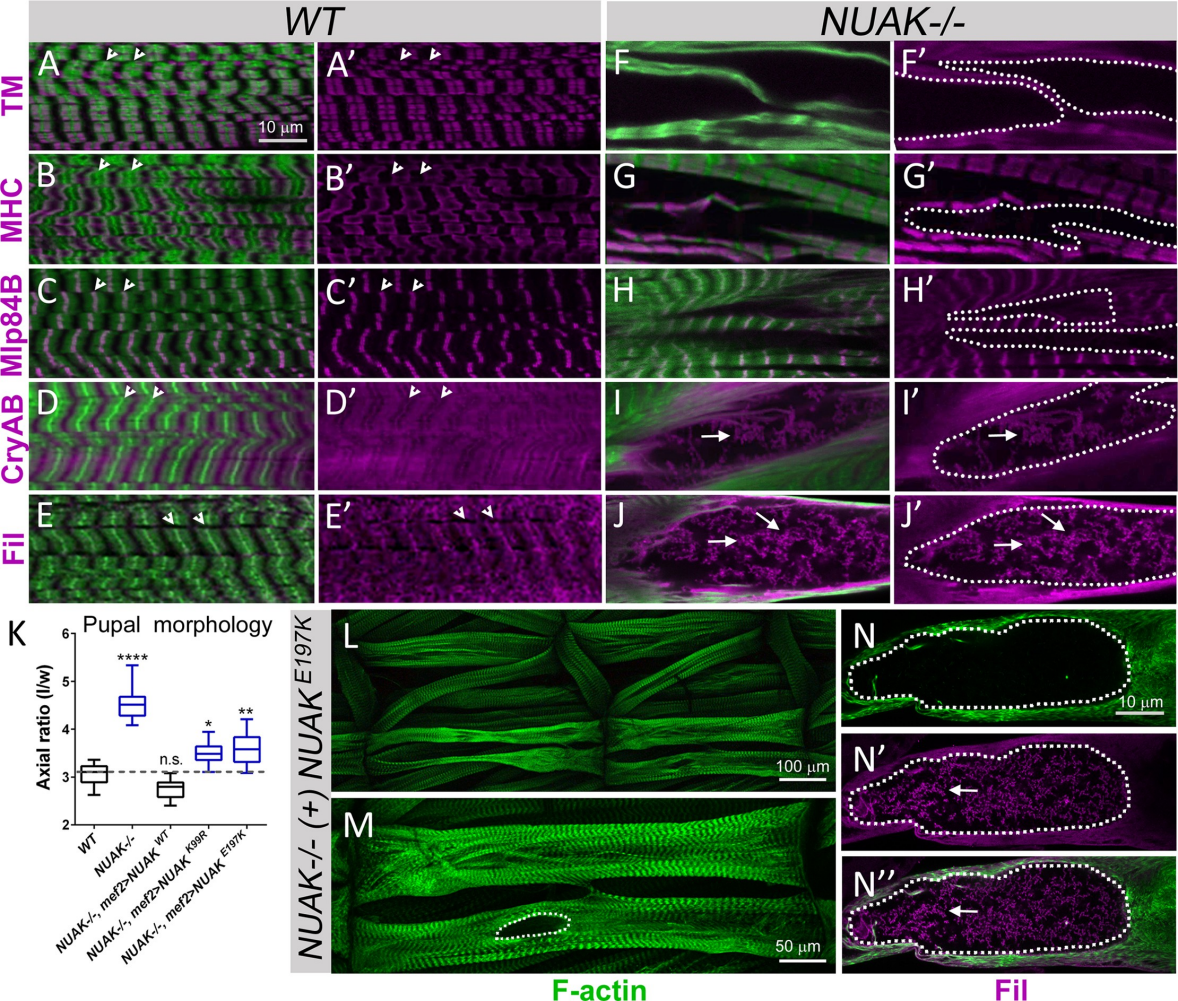
Select proteins accumulate in *NUAK-/-* muscles. (A-J’) Protein localization of F-actin (green) and the indicated proteins (purple) in *WT* (A-E’) or *NUAK-/-* (F-J’) L3 muscle. All images are cropped and represent internal regions of intact muscles. (A-E’) The normal localization of the thin filament protein TM (A,A’), the thick filament protein MHC (B,B’), or the Z-disc protein Mlp84B (C,C’) across multiple sarcomeres are shown. Both CryAB (D,D’) and Fil (E,E’) exhibit a broader localization in muscle tissue. Z-discs indicated by the white indented arrows. (F-J’) TM (F,F’), MHC (G,G’) and Mlp84B (H,H’) are all absent from regions that lack F-actin (white dotted outlines). In contrast, both CryAB (I, I’) and Fil (J,J’) can be found as aggregates (white arrows) in regions lacking F-actin (white dotted outlines) in the absence of NUAK. (K-N”) (K) Box and whisker plot of pupal case axial ratios of the indicated genotypes. Independent mutation of two residues in the kinase domain fail to rescue muscle contraction. *WT* axial ratios values are indicated the gray dashed line. Statistical values directly above each genotype are compared to *WT* control larvae. (L-N”) Phalloidin-labeled muscles (green) exhibit *NUAK*-like muscle phenotypes (white dotted outline) and the accumulation of Fil (purple). Mean +/- SD. (*, p<0.05; **, p<0.01; ****p<0.001; n.s., not significant).

To confirm the identity of proteins that correspond to the electron-dense aggregates in *NUAK-/-* muscle tissue, we immunostained L3 larvae with antibodies that label thin filament, thick filament, or Z-disc proteins. The actin-binding protein Tropomyosin (TM) is a thin filament protein that overlaps with F-actin adjacent to the Z-disc (**Fig 4A and 4A’**, Z-disc denoted by white indented arrowhead). In *NUAK-/-* muscles, TM was not present in regions that lacked phalloidin staining (**Fig 4F and 4F’**, white dotted lines). Similar results were obtained for the thick filament protein Myosin heavy chain (MHC). Whereas MHC alternated with F-actin in a periodic pattern in both *WT* (**Fig 4B and 4B’**) or *NUAK-/-* (**Fig 4G and 4G’**, white dotted lines) muscle tissue, there was no aberrant accumulation of MHC in other regions. Since TM and MHC did not accumulate in areas where sarcomeres were absent in *NUAK-/-* muscles, we next examined the localization of Z-disc proteins. Muscle LIM protein at 84B (Mlp84B) is found exclusively at the Z-disc in *WT* muscle (**Fig 4C and 4C’**) [33]. This Z-disc association was maintained in patterned regions within *NUAK-/-* muscle, but was absent in regions devoid of F-actin (**Fig 4H and 4H’**, white dotted lines). In contrast, the Z-disc proteins CryAB and Fil exhibited a different pattern. Both CryAB (**Fig 4D and 4D’**) and Fil (**Fig 4E and 4E’**) are present at the Z-disc (white indented arrowhead) with a broader distribution across the sarcomere in *WT* muscle [39]. CryAB (**Fig 4I and 4I’**) and Fil (**Fig 4J and 4J’**) were present in filamentous-like aggregates (white arrows) in *NUAK-/-* muscle, strongly suggesting that specific proteins accumulate in regions that lack F-actin structures.

Human NUAK1 has been shown to phosphorylate Myosin phosphatase target subunit 1 (MYPT) [40]. To determine if this catalytic activity is conserved in *Drosophila* NUAK, we generated transgenic flies with two independent kinase-dead mutations (K99R or E197K) [28, 41]. Recombination of these mutations into a *NUAK-/-* background failed to rescue muscle contraction during the larval to pupal transition (**Fig 4K**). Further analysis of the E197K mutation revealed muscle degeneration similar to those observed in *NUAK* mutants (**Fig 4L–4N”**) with an accumulation of Fil in regions devoid of F-actin (**Fig 4N–4N”**, white dotted lines). These results show that NUAK kinase activity is important in preventing muscle degeneration and the abnormal accumulation of Fil protein.

Control experiments were performed to confirm that the accumulation of select proteins upon loss of NUAK is indeed due to inherent defects inside each myofiber. First, intentional damage to *WT* muscles did not show an accumulation of Fil in regions lacking phalloidin staining (**S4 Fig,** indented arrowheads). Second, the sarcolemma was still intact in *NUAK-/-* muscles (**S4 Fig**), ruling out internal protein loss due to damaged membranes. In conclusion, our TEM and immunostaining analysis shows that loss of NUAK results in the selective accumulation of a subset of muscle proteins.

### NUAK biochemically interacts with Stv/BAG3 and Fil

**Fig 5:**
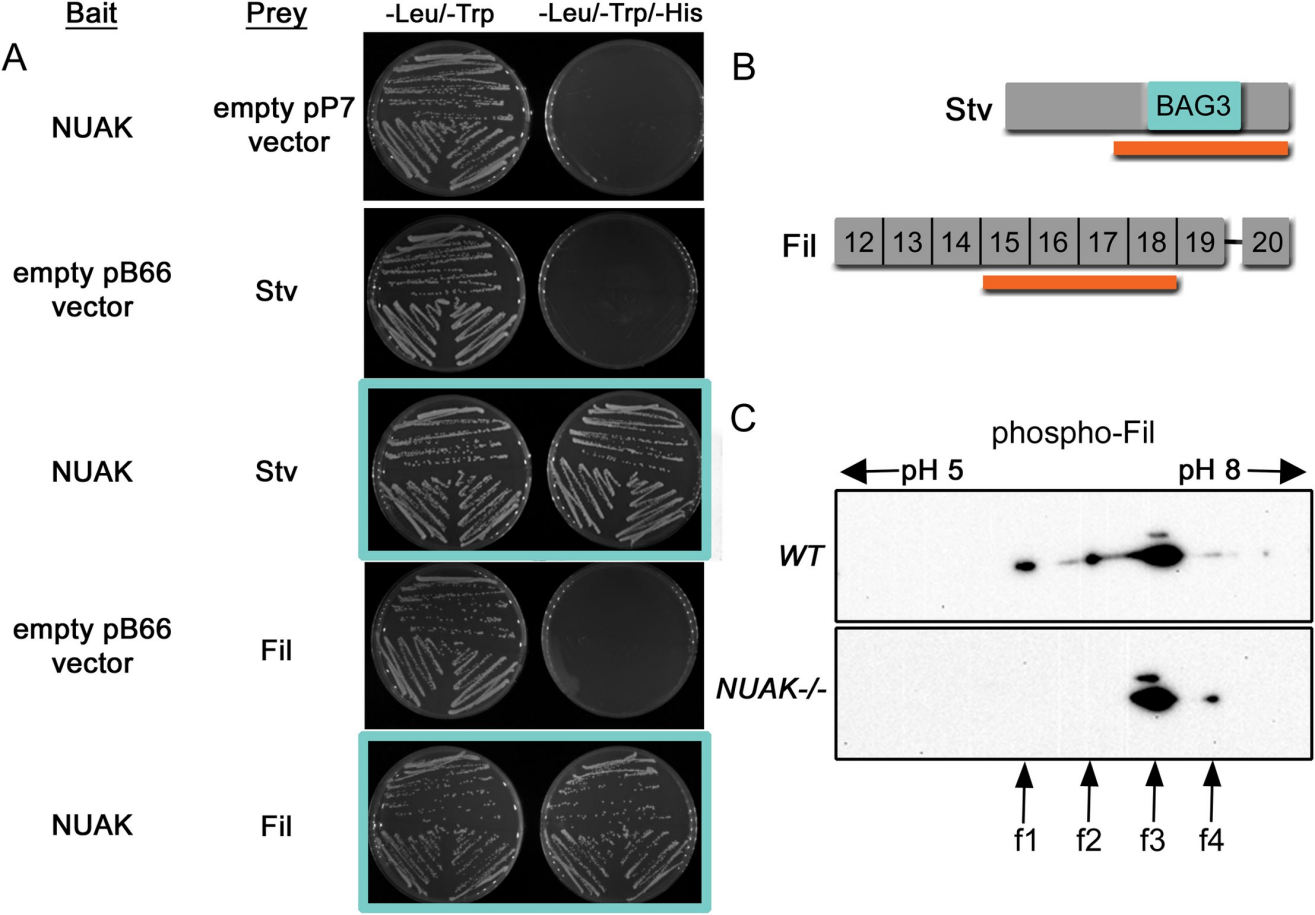
Stv/BAG-3 and Fil physically interact with NUAK. (A) Confirmation of one-by-one Y2H interaction results using NUAK as a bait with Stv and Fil as prey proteins. The selective medium lacking tryptophan and leucine is used as a positive control to verify the presence of the bait and prey plasmids. Three independent yeast clones were streaked onto plates lacking histidine to verify NUAK protein interactions with Stv or Fil (blue boxes). (B) Schematic of *Drosophila* Stv and the C-terminal region of Fil isoforms (Ig domains 12–20). The orange bar represents the selected interaction domain of NUAK with the conserved BAG3 domain of Stv and Ig domains 15–18 in Fil. (C) 2D gel Western blot of *WT* or *NUAK-/-* muscle carcasses probed with anti-Fil. Two forms of phosphorylated Fil in *WT* muscle (f1 and f2) disappear upon loss of NUAK. A predominant spot likely corresponding to non-phosphorylated Fil (f3) is present in both *WT* and *NUAK-/-* muscle tissue, with f4 present as a possible minor form.

A role for NUAK in muscle degeneration and/or protein aggregation has not been reported. Therefore, we chose a yeast two-hybrid screening approach (Y2H) to gain an unbiased molecular understanding of NUAK function. Full length *Drosophila* NUAK (AA1- 1180) was cloned in-frame with the Gal4 DNA binding domain and this bait was utilized to screen a *Drosophila* L3 library. Three clones corresponding to Stv and twenty-nine clones encoding for Fil emerged as prey proteins. A clone for each (A-255 and A-105) was further selected for validation. After independent retransformation of both bait and prey vectors, we confirmed a direct, physical interaction between NUAK with Stv or Fil (**Fig 5A**). Analysis of Stv prey fragments that bind to NUAK reveal that the interaction domain encompasses amino acids 322–516, which includes the conserved BAG domain (**Fig 5B**). Ig domains 15–18 of Fil were found to interact with NUAK. Since phosphorylated forms of Filamin A and Filamin C have been identified in mammalian muscle tissue [42–44], we posited that *Drosophila* Fil may be a substrate for NUAK kinase activity. To test this possibility, we looked for differences in the relative migration of Fil protein after 2D gel analysis followed by Western blotting. Intriguingly, the migration pattern of Fil differed between *WT* and *NUAK-/-* samples. We identified four distinct spots corresponding to modified or unmodified forms of Fil (f1-f4) (**Fig 5C**). While the position of spots f3 and f4 appeared similar in *WT* and *NUAK-/-* muscle tissue, the prevalent f1 and f2 spots shifted towards a positive pI upon loss of NUAK, which we assume is due to loss of negatively-charged phosphate group(s) on Fil.

**Fig 6:**
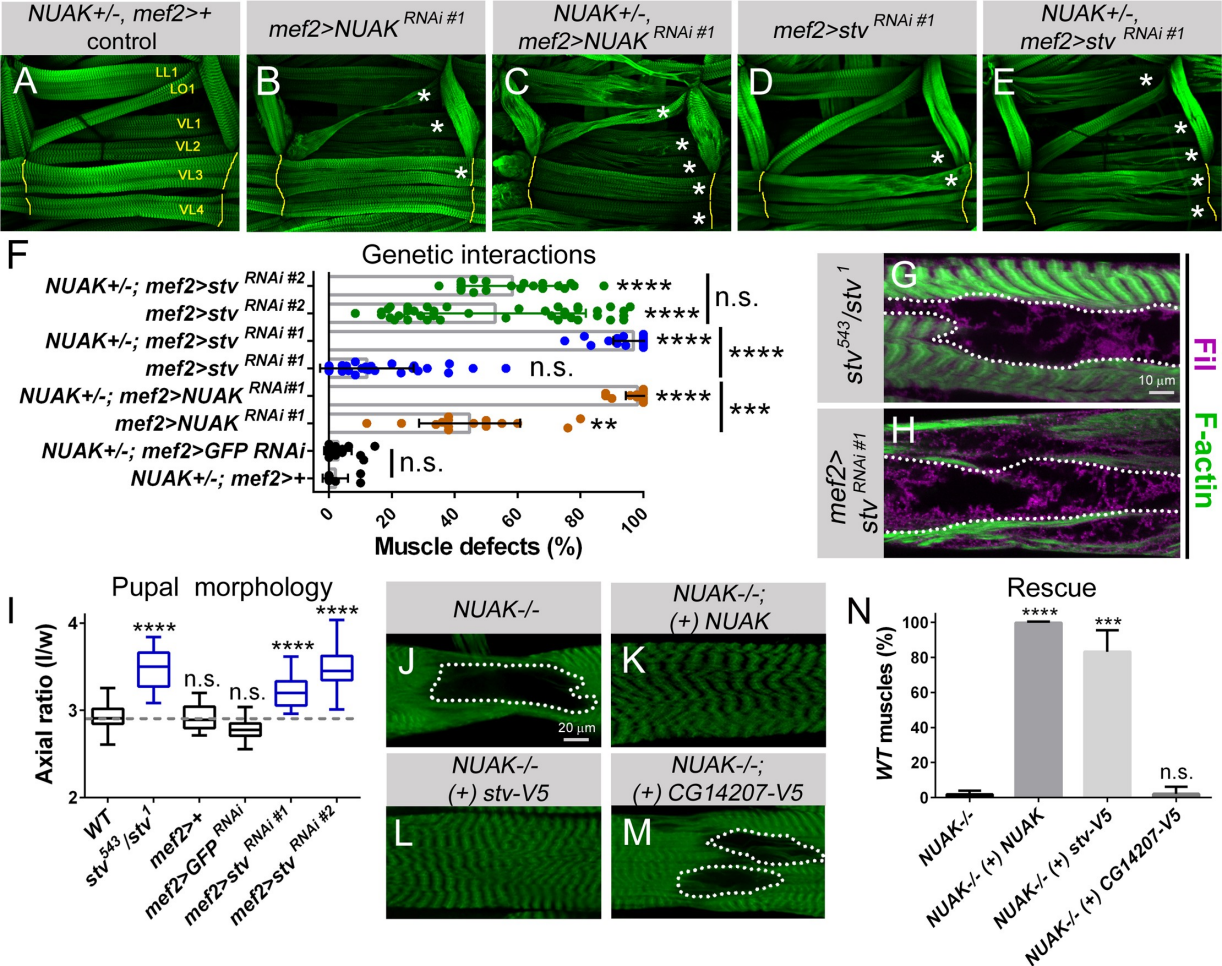
*NUAK* and *stv* genetically interact in muscle tissue. (A-E) Muscles LL1, LO1, and VL1-4 in one hemisegment of the L3 musculature stained for F-actin (green). *NUAK +/-*, *mef2-*Gal4 muscles appear *WT* at 25°C. (B) Weak muscle defects are prevalent upon muscle expression of *UAS-NUAK*^*RNAi #1*^ alone. (C) This same reduction in *NUAK* transcript levels enhances muscle phenotypes in a heterozygous *NUAK+/-* background. (D,E) Weak perturbations in muscle morphology upon induction of UAS-*stv* ^*RNAi #1*^ alone (D) are enhanced upon removal of one copy of *NUAK* (E). (F) Scatter and bar plot depicting the percentage of muscle defects in the indicated genotypes. (G,H) Fil (purple) accumulates in regions lacking F-actin (green) in *stv*^*543*^*/stv*^*1*^ mutants (G) or upon muscle-targeted expression of *stv RNAi* (H). (I) Box and whisker plot of the indicated genotypes. A decrease in *stv* using mutant alleles (*stv*^*543*^*/stv*^*1*^) or through the induction of two independent RNAi lines (*UAS-stv*^*RNAi #1*^ or *UAS-stv*^*RNAi #2*^) both exhibit defects in muscle contraction, resulting in an extended pupal axial ratio. *WT* values are indicated by gray dashed line. Statistical values directly above each genotype are compared to *WT* control larvae. (J-M) F-actin-labeled VL3 muscle (green). Areas that lack phalloidin staining (white dotted outlines) in *NUAK-/-* (J) are rescued upon the reintroduction of full length *NUAK cDNA* in muscle tissue (*NUAK-/-; mef2>NUAK*) (K). Expression of a V5-tagged Stv protein rescues *NUAK-/-* phenotypes (L), while overexpression of CG14207-V5 fails to rescue (M). (N) Bar graph depicting rescue results. Mean +/- SEM. (**, p<0.01; ***, p<0.005; ****, p<0.001; n.s., not significant).

We next wanted to confirm an in vivo role for the NUAK-Stv complex in muscle tissue to functionally verify our Y2H interaction. We leveraged the power of the temperature-dependent Gal4/UAS system [45] to develop a muscle-specific genetic interaction assay in which six muscles in each hemisegment [longitudinal lateral muscle 1 (LL1), lateral oblique 1 (LO1), VL1-4] were evaluated for muscle morphology defects. Muscles that were heterozygous for *NUAK* and a muscle Gal4 driver (*NUAK+/-; mef2>+*) appeared normal and show that a single *WT* copy of *NUAK* is sufficient to maintain L3 muscles (**Fig 6A and 6F**). At 25°C, a temperature with intermediate Gal4 expression, induction of the weaker NUAK RNAi construct (*mef2>NUAK RNAi #1*) resulted in morphological defects in ~40% of muscles analyzed (**Fig 6B and 6F**). However, this same reduction in *NUAK mRNA* levels in a heterozygous *NUAK+/-* background increased the percentage of affected muscles to nearly 100% (**Fig 6C and 6F**). Thus, a NUAK sensitized background was successfully established to evaluate genetic interactions between *NUAK* and *stv*.

Two independent *stv RNAi* lines that reduced *stv* transcript levels more than 50% (**S5 Fig**) were induced under control of the *mef2* promoter. Each line showed variable defects ranging from 20–50% of muscles affected in each hemisegment (**Fig 6F, S5 Fig**). A representative example of the morphological phenotypes seen upon muscle-specific expression of the weaker *stv RNAi* line #1 is shown in **Fig 6D**. Knockdown of this same *stv RNAi* line in a heterozygous *NUAK+/-* background enhanced the percentage of defective muscles (**Fig 6E and 6F**). Note that induction of the stronger *stv RNAi* line #2 did not significantly enhance muscle defects in *NUAK+/-* larvae (**Fig 6F**), likely because knockdown of *stv* transcript was already reduced enough to cause severe muscle defects. No muscle abnormalities were observed upon induction of an exogenously supplied *GFP RNAi* in a NUAK-sensitized background (similar to the *NUAK+/-; mef2>+* control) (**Fig 6F**). These data demonstrate a genetic interaction between *NUAK* and *stv* in muscle tissue, further supporting our Y2H interaction data.

Individuals homozygous for *stv*^*1*^ are lethal before the end of the first larval instar (L1) stage [11, 46]. To examine a role for Stv in L3 muscle maintenance, we examined the partially lethal P-element insertion allele *stv*^*00543*^ in combination with *stv*^*1*^ *(stv*^*00543*^/*stv*^*1*^*)*. Muscles of this genotype recapitulated *NUAK-/-* phenotypes, including the accumulation of Fil in regions devoid of F-actin (dotted lines in **Fig 6G**). We further confirmed *NUAK*-like muscle and Fil aggregation phenotypes upon muscle-targeted expression of *stv RNAi* (**Fig 6H, S5 Fig**). These morphological defects correlate with muscle dysfunction as *stv* mutants or *stv RNAi* knockdown larvae failed to contract their musculature during pupal morphogenesis. The pupal axial ratio for all genotypes with reduced *stv* levels is significantly longer than *WT* or *mef2>*+ driver controls (**Fig 6I**). Thus, partial loss of Stv using hypomorphic allelic combinations or RNAi techniques phenocopies *NUAK* muscle defects, further supporting our conclusion that *NUAK* and *stv* genetically interact.

We next utilized a genetic overexpression approach to determine the relationship between *NUAK* and *stv*. Regions lacking F-actin in *NUAK-/-* muscle (**Fig 6J**) were rescued to *WT* muscle morphology upon expression of a full length *NUAK cDNA* (**Fig 6K and 6N**). Similar results were observed upon overexpression of a V5-tagged version of Stv (**Fig 6L and 6N**), while induction of an independently tagged chaperone (CG14207-V5) failed to ameliorate these phenotypic abnormalities (**Fig 6M and 6N**). Thus, Stv functions parallel to or downstream of NUAK. To test if NUAK can function downstream of Stv, we performed a reciprocal type of rescue experiment. While over-expression of NUAK is sufficient to rescue muscle defects in a *NUAK RNAi* background, this same NUAK over-expression failed to rescue *stv*-mediated muscle defects (**S6 Fig**). These data collectively show that NUAK and Stv not only biochemically interact, but also function within the same genetic pathway to prevent muscle degeneration.

### NUAK and Stv are required for the autophagic degradation of Fil

**Fig 7:**
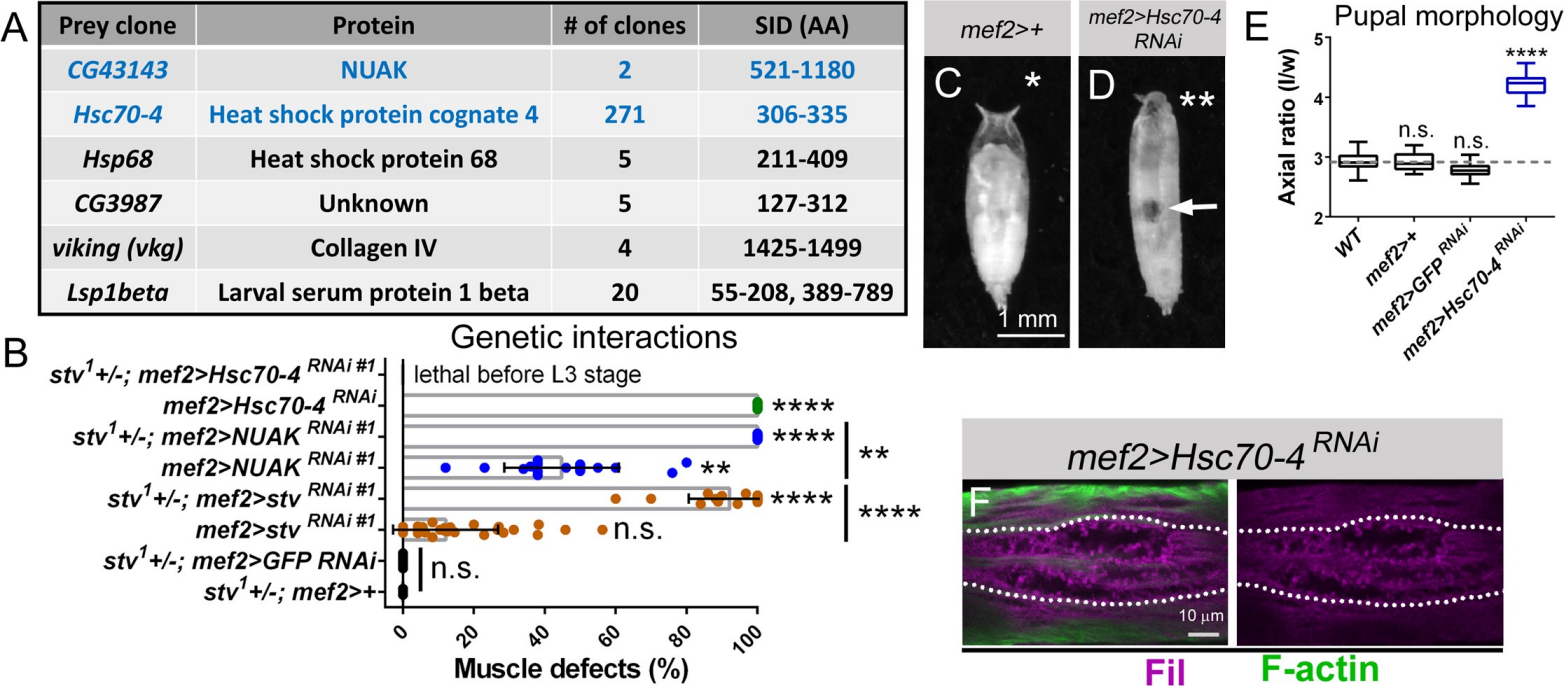
Hsc70-4 is required to prevent protein aggregation. (A) Table showing prey proteins that interact with Stv in a Y2H screen. SID = selected interaction domain. (B) Scatter and bar plot demonstrating a genetic interaction between *stv* and *NUAK*. RNAi knockdown of *NUAK* or *stv* further enhances the percentage of muscle defects in a heterozygous *stv* genetic background (*stv*^*1*^ *+/-*). (C,D) Pupal case morphology. (C) Flies that contain an insertion of the *mef2*-Gal4 driver appear *WT*. (D) RNAi knockdown of *Hsc70-4* causes an elongated pupal case, defective spiracles (double asterisks) and a failure to displace the abdominal air bubble (arrow) due to defective muscle contraction. (E) Quantification of pupal axial ratios of the indicated genotypes clearly show that decreased *Hsc70-4 mRNA* levels exhibit muscle contraction defects compared to *WT* or *mef2* controls (gray dashed line). (F) Muscles of the genotype *mef2>Hsc70-4 RNAi* show accumulation of Fil (purple) in areas that lack F-actin (green). Mean +/- SEM. (**, p<0.01; ****, p<0.001; n.s., not significant).

Stv is the *Drosophila* ortholog of mammalian BAG3, a molecular co-chaperone implicated in numerous biological processes, including apoptosis, development, cytoskeletal dynamics, and autophagy [16, 47–49]. Using full length Stv as a bait, we again screened an L3 library using the Y2H approach. High confidence interactions are listed in **Fig 7A**. Two clones of NUAK were identified, further verifying the Stv-NUAK physical interaction. The highest number of clones encoded for Heat shock protein cognate 4 (Hsc70-4). BAG3 is well established as a nucleotide-exchange factor for the HSC70 ATPase that promotes the release of ADP and associated client proteins [50–52]. To confirm the Stv-NUAK and Stv-Hsc70-4 interactions in muscle tissue, we postulated that a heterozygous *stv* background may be useful for detecting genetic interactions. Larval muscles heterozygous for the *stv*^*1*^ allele [46] recombined with *mef2*-Gal4 (*stv+/-*, *mef2>*) alone or crossed to *GFP RNAi* appeared normal (**Fig 7B**). Consistent with our data showing that *NUAK* and *stv* function together (**Fig 6F**), muscle defects were increased when the mRNA levels of *NUAK* were reduced in larvae containing a single copy of *stv* (**Fig 7B**). We next examined if RNAi knockdown of Hsc70-4 also enhanced muscle phenotypes in a heterozygous *stv+/-* background. However, induction of *Hsc70-4 RNAi* alone resulted in 100% defective muscles (**S7 Fig**) and larvae did not survive until the L3 stage in a sensitized *stv* background (**Fig 7B**). Even at the low temperature of 18°C, *mef2>Hsc70-4 RNAi* individuals failed to contract their body wall muscles during the larval to pupal transition (**Fig 7C–7E**) and showed Fil accumulation in regions lacking F-actin (**Fig 7F**). Here we confirm that Hsc70-4 is a binding partner of Stv and conclude that a decrease of Hsc70-4 in muscle phenocopies the aggregation and cellular degeneration defects in *NUAK* or *stv* mutants.

**Fig 8:**
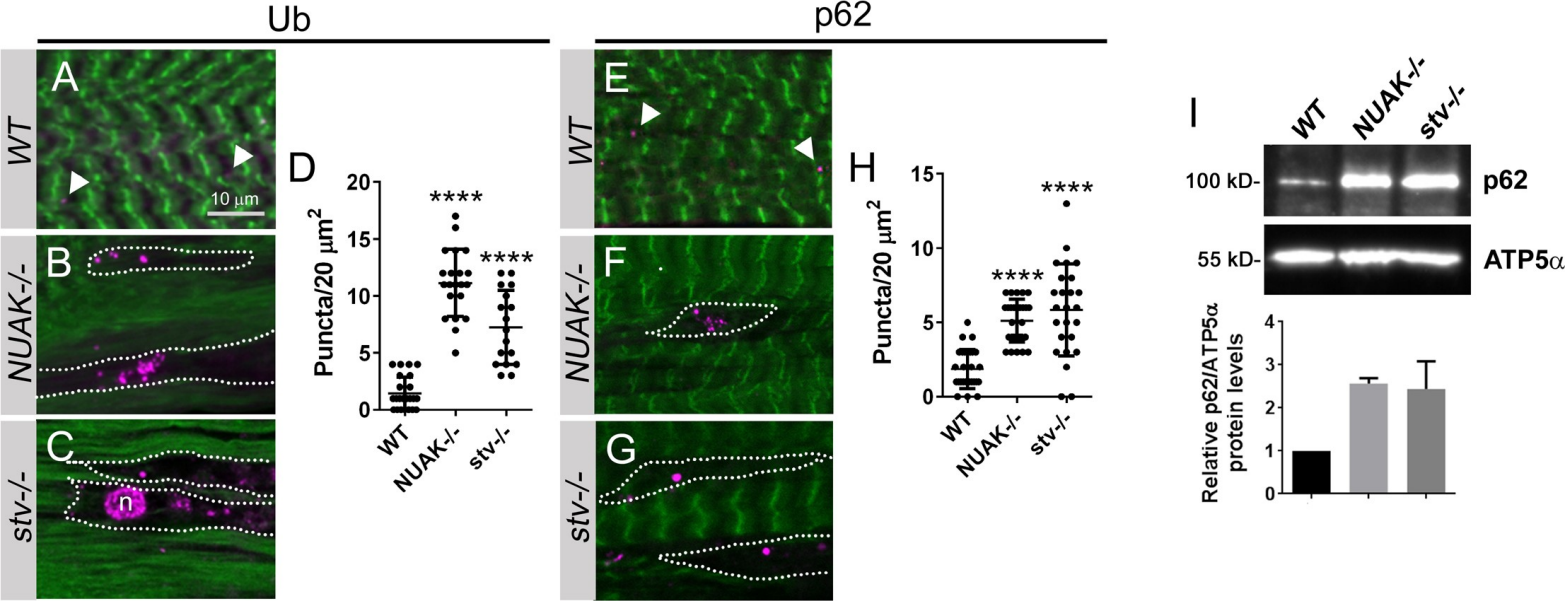
Autophagy is blocked upon loss of the NUAK-Stv complex. (A-C,E-G) Anti-Ub (A-C) or anti-p62 (E-G) immunostaining (purple) in L3 muscle tissue (green, F-actin). (A) Ub-(+) puncta (white arrowheads) are occasionally present in *WT* muscle. (B,C) Puncta that stain positive for Ub are clustered in areas of the muscle where F-actin is excluded (white dotted outlines) in *NUAK-/-* (B) or *stv-/-* (C). (D) Scatter plot depicting the number of Ub(+) puncta/20 um^2^. (E) Similar to Ub, puncta corresponding to p62 (white arrowheads) are found in normal muscle tissue. (F,G) p62(+) puncta are present in greater numbers upon loss of NUAK (F) or Stv (G). (H) Quantitation of p62(+) puncta/20 um^2^ depicted by a scatter plot. (I) Western blot of whole L3 larvae reveals a block in autophagy indicated by elevated p62 protein levels in *NUAK* or *stv* mutants. ATP5α is used as a loading control. Bar graph depicts the ratio of p62/ ATP5α intensity in the indicated genotypes. N = 3. (Mean +/- SEM. (*, p<0.05). Mean +/- SEM. (****, p<0.001).

Aggregation-prone client proteins, such as Fil, are recognized by a multi-chaperone complex consisting of Stv/BAG3 and Hsc70-4/Hsc70 to induce ubiquitination and p62/SQSTM1 recruitment [6, 7, 16, 47, 49]. Because Fil abnormally accumulates in *NUAK-/-* and *stv-/-* muscle tissue, we hypothesized that both Ub and p62 may also associate in these regions. Antibodies that detect either Ub or p62 moieties were utilized to examine their distribution in *WT* or mutant muscle tissue. Puncta corresponding to Ub (**Fig 8A**, white arrowhead) were occasionally present in *WT* muscle, but more numerous in *NUAK-/-* (**Fig 8B and 8D**) or *stv-/-* (**Fig 8C and 8D**) muscle tissue. Similarly, p62(+) puncta were observed at low numbers in normal muscle (**Fig 8E**), but accumulated in regions lacking F-actin in *NUAK-/-* (**Fig 8F and 8H**) or *stv-/-* (**Fig 8G and 8H**). This increased number of p62 puncta strongly suggests that autophagy is impeded upon loss of NUAK as has been reported for BAG3 [6, 16, 49]. Indeed, Western blot analysis confirmed that the overall levels of p62 were elevated in *NUAK* or *stv* mutant larvae (**Fig 8I**). Hence, we conclude that a nonfunctional NUAK-Stv complex blocks autophagic protein degradation, with a corresponding accumulation of Ub and p62.

**Fig 9:**
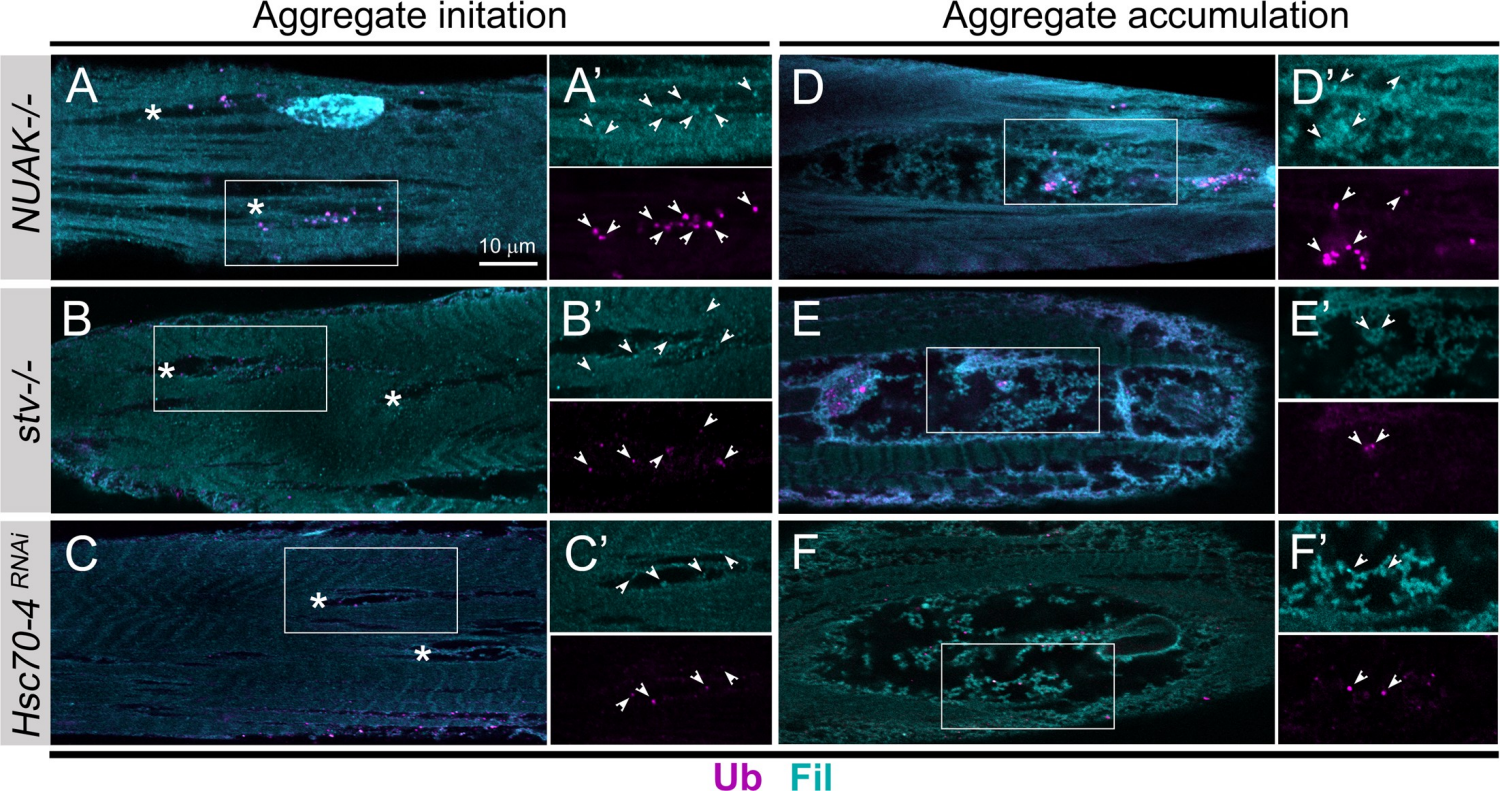
Ub distribution changes during aggregate accumulation. (A-F) Fil (blue) and Ub (purple) immunostaining in L3 muscles in the early (A-C’) or late (D-F’) stages of aggregate accumulation. A decrease in NUAK (A,A’), Stv (B,B’), or Hsc70-4 (C,C’) results in small regions of muscle tissue that begin to accumulate puncta decorated with Fil and Ub puncta (white indented arrowheads). In large areas of aggregate accumulation in *NUAK-/-* (D,D’) and *stv-/-* (E,E’) mutants or upon *Hsc70-4 RNAi* knockdown (F,F’), the co-localization of Ub(+) puncta with Fil protein (white indented arrowheads) is decreased. The white boxed regions in A-F are enlarged in A—F’.

While there is an increase in the number of Ub puncta, the location of these Ub molecules do not fully recapitulate the abnormal pattern of Fil immunostaining in *NUAK* (**Fig 4J and 4J’**) or *stv* (**Fig 6G**) mutants. Thus, we decided to compare Fil and Ub distribution in small regions that begin to show muscle deterioration (initiation) with larger areas in which the accumulation of select proteins has already occurred (aggregation). Puncta that stain positive for both Ub and Fil (white indented arrowheads) were abundant in areas just beginning to show changes in muscle morphology upon a reduction in NUAK (**Fig 9A and 9A’**), Stv (**Fig 9B and 9B’**), or Hsc70-4 (**Fig 9C and 9C’**). However, in large regions that exhibit atypical Fil accumulation, only a subset of Fil protein was decorated with Ub (white indented arrowheads) upon perturbation of NUAK (**Fig 9D and 9D’**), Stv (**Fig 9E and 9E’**), or Hsc70-4 (**Fig 9F and 9F’**). These similar patterns of Fil and Ub colocalization suggest a common mechanism whereby Fil molecules are initially marked by poly-Ub and a failure to clear these Fil-Ub complexes results in heterogeneous aggregate formation.

**Fig 10:**
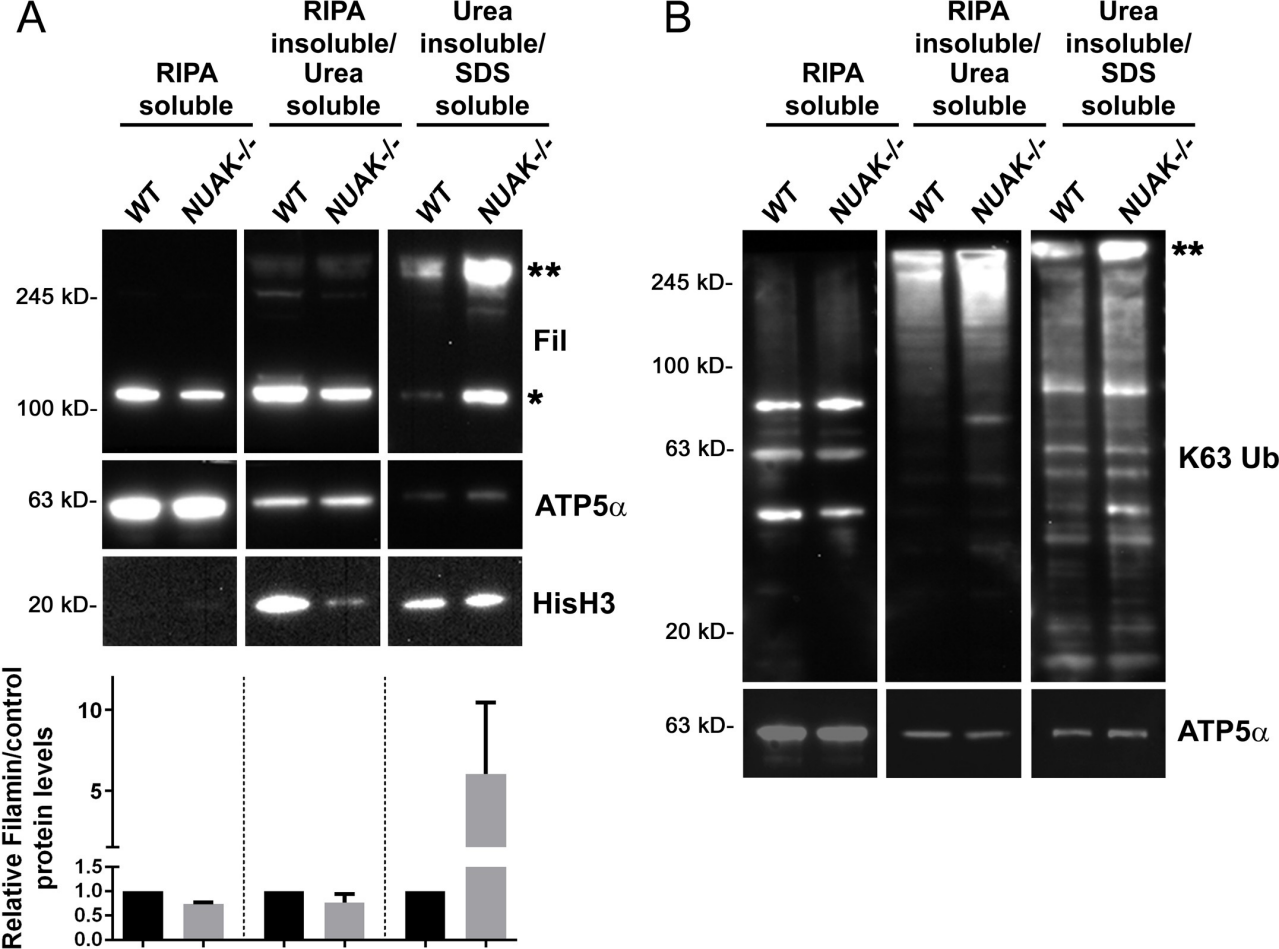
Loss of NUAK results in the insoluble accumulation of Fil and K63-linked Ub. (A,B) Western blots probed for Fil (A) or K63-linked Ub chains (B) after fractionation of *WT* or *NUAK-/-* L3 muscle carcasses into soluble or insoluble fractions. (A) Fil protein levels are similar in RIPA or Urea-soluble fractions, but preferentially increased in the pellet (solubilized by SDS, single asterisk). A substantial portion of aggregated Fil protein did not migrate into the gel (double asterisks). Densiometric quantitation of Fil protein levels (single asterisk) reveals ~5-fold increase in Fil protein levels upon loss of NUAK. (B) Poly-Ub chains that contain K63 linkages broadly accumulate in the insoluble fractions of *NUAK-/-*. Similar to Fil, a portion of K63-linked proteins accumulate in the top of the SDS-PAGE gel (double asterisk). The loading control ATP5α is largely soluble, but also present in Urea and SDS-soluble fractions. HisH3 was used to confirm insoluble loading.

Multiple pieces of evidence thus far suggest that protein aggregates accumulate in *NUAK*-/-muscle tissue: (1) TEM analysis shows that electron-dense protein aggregates replace myofibrillar material; (2) heterogeneous aggregate-like structures of Fil and CryAB are observed by immunofluorescence in regions that lack F-actin; and (3) there is an increased number of puncta corresponding to Ub that colocalizes with Fil protein. To show that Fil is indeed present in insoluble aggregates, we performed biochemical fractionation on *WT* or *NUAK-/-* muscle carcasses followed by Western blotting. Fil was found in the RIPA and Urea soluble fractions of both *WT* and *NUAK-/-* lysates in approximately equal amounts (**Fig 10A**). However, upon loss of NUAK, large amounts of insoluble Fil were present at the expected molecular weight (single asterisk) and in a high molecular weight species that failed to enter the gel (double asterisk). Aggregates linked to K63-based ubiquitin chains are typically found in insoluble fractions when autophagy is blocked [2, 53, 54]. Using an antibody specific for K63-linked ubiquitin chains, we confirmed enrichment of this poly-ubiquitinated species in the insoluble fractions of *NUAK-/-*muscle tissue (**Fig 10B**). Notably, a large amount of K63-linked proteins was also present at the top of the gel (double asterisk), similar to that for Fil. These experiments importantly demonstrate that Fil abnormally accumulates in insoluble aggregates.

**Fig 11:**
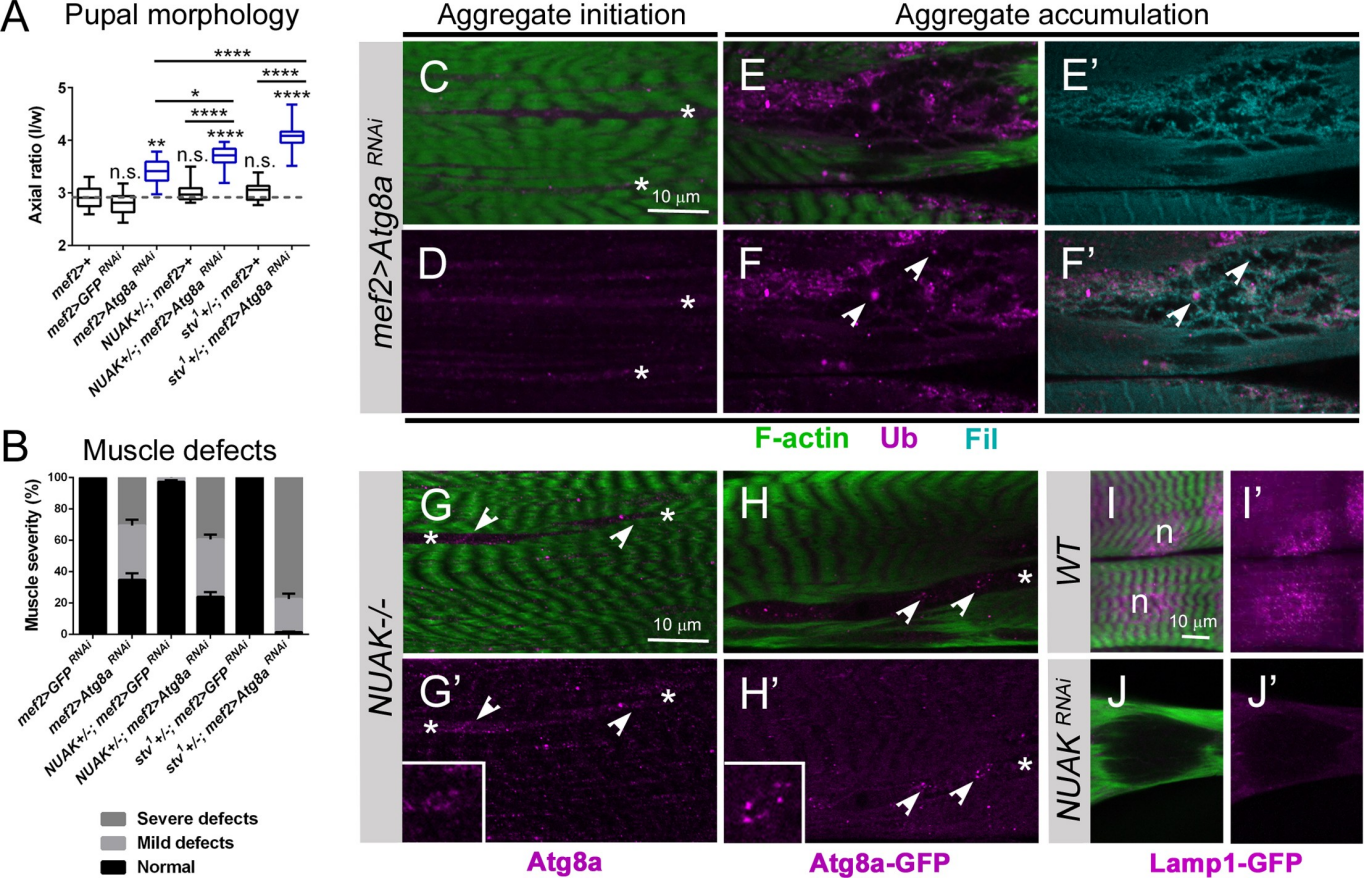
*Atg8a* genetically interacts with *NUAK* and *stv* and is required to prevent protein aggregation. (A) Axial ratios as a measure of muscle contraction are represented by a box and whisker plot. Gray dashed line indicates *WT* and control values. Statistical values directly above each genotype are compared to *WT* control larvae. (B) A grouped column plot shows the relative severity of muscle aggregation defects in *Atg8a RNAi* alone that are enhanced upon removal of one copy of *stv*. Regions that showed initiation of abnormal muscle morphology were considered mild defects and large aggregate holes were classified as severe defects. (C-F’) Ub (purple) or Fil (blue) immunostaining in *mef>Atg8aRNAi* muscle tissue labeled with phalloidin (green). (C,D) In small regions where F-actin is displaced (asterisks), Ub accumulation is observed. (E-F’) In larger regions where F-actin is missing, Ub puncta can be found decorating Fil protein aggregates (white indented arrowheads). (G-H’) Puncta corresponding to Atg8a (purple) accumulate in regions lacking F-actin (asterisks). Atg8a puncta detected by immunostaining (G,G’) or visualized with a GFP fusion protein (H,H’) can be found in ring-like structures (insets). (I-J’) Lamp1-GFP puncta (purple) are observed in the perinuclear region in *WT* muscle tissue, but cannot be found in regions lacking F-actin upon loss of NUAK (J,J’). Mean +/- SEM. (****, p<0.001; not significant).

The core autophagy protein Atg8a/LC3 is recruited by p62 and is required for the biogenesis of autophagosomal membranes for eventual protein disposal in the lysosome [55, 56]. *Drosophila* possesses two *Atg* genes, *Atg8a* and *Atg8b*. *Atg8b* expression is high is adult testes and weakly expressed in larval fat body tissue [57, 58]. Thus, we next examined a genetic role for the ubiquitously expressed *Atg8a* in preserving muscle function. Muscle-specific *RNAi* silencing of *Atg8a* impaired muscle contraction during the larval to pupal transition (**Fig 11A**). The axial ratio of these *mef2>Atg8a RNAi* pupal cases was enhanced upon removal of a single copy of *NUAK* or *stv*. As expected, the heterozygous *NUAK+/-; mef2>+* or *stv+/-; mef2>+* pupal cases were similar to *mef2-Gal4* or *mef2>GFP RNAi* controls. *mef2>Atg8a RNAi* muscles showed thinning muscles with regions devoid of F-actin (**S7 Fig**). The penetrance of these muscle defects (~60% in *mef2>Atg8a* RNAi alone) remained the same in the *NUAK+/-* background, but was enhanced to almost 100% in a heterozygous *stv* background (**Fig 11B**).

Next we assessed Ub or Fil distribution in muscles with reduced Atg8a. Ub (+) puncta were present in regions starting to lose normal F-actin morphology (**Fig 11C and 11D**, asterisks). In large aggregate regions, we observed Fil accumulation decorated with Ub protein (**Fig 11E–11F’,** white indented arrowheads). Atg8a protein, assayed by immunostaining (**Fig 11G and 11G’**) or through visualization of an Atg8a-GFP fusion protein (**Fig 11H and 11H’**), was confirmed in regions lacking F-actin (asterisks). Moreover, some of these Atg8a(+) puncta appeared to be organized in ring-like structures, indicative of autophagosome formation (inset in **Fig 11G’ and 11H’**). Since the fusion of autophagosomes with lysosomes is required for cargo clearance, we next assessed whether lysosomes were present in aggregate regions. Visualization of lysosomes with Lamp1-GFP revealed a normal perinuclear accumulation in *WT* muscle (**Fig 11I and 11I’**). However, Lamp1-GFP was never observed in regions of aggregate accumulation upon loss of NUAK (**Fig 11J and 11J’).** These results suggest that Atg8a is recruited to form autophagosomes, but a failure to undergo lysosomal fusion prevents client protein turnover in *NUAK-/-* muscle tissue.

## Discussion

Here we identify a novel protein aggregation phenotype in *NUAK*-/- muscle tissue that directly impacts sarcomere morphology and contractile function. Our data conclusively show that NUAK phosphorylates Fil and functions with Stv/BAG3 in autophagy-mediated protein clearance. The additional requirement of the HSC70 family member Hsc70-4/HSPA8 with Stv further substantiates a role for these proteins in autophagic protein turnover.

### NUAK regulation of myofilament and cytoskeletal proteins

Prior to our study, few substrates of NUAK kinase activity had been uncovered. One of these is Myosin phosphatase targeting-1 (MYPT1), a regulatory subunit of myosin light-chain phosphatase [40]. We tested two *Drosophila* regulatory subunits, MYPT75D and Myosin binding subunit (Mbs) [59, 60] in our *NUAK* sensitized genetic assay and failed to observe protein aggregation and/or muscle degeneration. While negative, this data nevertheless argues that this family of phosphatases likely does not function with NUAK in muscle tissue. Since the mammalian NUAK1-MYPT1 interaction was identified in vitro and further validated in HEK293 cells, NUAK likely has cell and tissue-specific targets that regulate diverse biological outputs.

Based upon our discovery of Fil as a novel NUAK substrate (**Fig 5C**), we envision two scenarios that are not mutually exclusive to explain the molecular function of NUAK in preventing protein aggregation. First, the increase in sarcomere number upon muscle-specific *NUAK RNAi* (**Fig 2J and 2L**) suggests that at least one role of NUAK may be to negatively regulate the addition of proteins (such as Fil) into sarcomeres. This data is consistent with studies that show *C*. *elegans* Unc-82 regulates myofilament assembly [28, 29]. Notably, one key feature of the misincorporated proteins in *unc-82* mutants is their inclusion into aggregate-like structures, similar to the accumulation of Fil and CryAB in *NUAK-/-* muscles. An additional, or alternative possibility, is that NUAK phosphorylates unfolded or ‘damaged’ Fil for removal from the sarcomere, thereby triggering the Stv-Hsc70-4 complex to promote autophagic turnover. Thus, proteins such as Fil that fail to get incorporated into sarcomeres and/or sustain damage due to repeated rounds of tension-induced muscle contraction, may destabilize myofilament architecture and trigger abnormal protein aggregation.

### The NUAK-BAG3 pathway

In both contractile muscle tissue and in adherent cells subjected to mechanical force, BAG3 acts as a hub to coordinate Fil-induced tension-sensing and autophagosome formation [6, 7, 16, 47]. The MSR of Fil is comprised of Ig repeats whose conformational transitions between open and closed states dictate differential protein-protein interactions and biological outputs [11, 61, 62]. While the chaperones Hsc70/HSPA8 and HSPB8 weakly bind to the MSR of Fil, this biochemical interaction is greatly enhanced in the presence of BAG3 [12]. Interestingly, BAG3 interacts with Ig repeats 19–21 in the MSR, while the selected interaction domain of NUAK with Fil comprises Ig repeats 15–18 (**Fig 5B**). These data suggest that NUAK and Stv each bind to a separate region of the MSR in Fil.

It remains to be determined if NUAK-mediated phosphorylation is a prerequisite for the removal of damaged Fil protein by BAG3 [10, 11]. Our rescue results suggest that this phosphorylation event is not required as Stv overexpression alleviates protein aggregation and muscle degeneration upon a loss of NUAK (**Fig 6L and 6N**). An alternative possibility is that this excess Stv protein is present in sufficient amounts to interact with Fil and overcome the necessity for phosphorylation by NUAK. The inability of NUAK overexpression to restore muscle defects due to knockdown of Stv, Hsc70-4, or Atg8a (**S6 Fig**) suggests that NUAK functions upstream or parallel to this pathway. It seems likely that NUAK has additional target substrates for kinase activity that may regulate autophagic protein clearance in muscle tissue.

Recent studies demonstrate that increased autophagic degradation of Fil by BAG3 also induces *fil* transcription as a compensatory mechanism to ensure steady-state Fil levels. Thus, we tested whether loss of NUAK or Stv alters gene expression upon a block in protein clearance. While the *mRNA* levels of *cher*, *CryAB*, *Hsc70-4*, or *Atg8a* were not altered in *NUAK* or *stv* mutants, there was a large increase in *p62* transcripts (**S8 Fig**). Thus, this increase in *p62 mRNA* synthesis may contribute to the elevated p62 protein levels observed upon loss of NUAK or Stv as multiple stress conditions increase p62 transcription, including proteasome inhibition, starvation and atrophic muscle conditions [63, 64]. Data that support a role for an autophagic block include the localization of p62 and Atg8a to regions of protein aggregation.

### Model for NUAK function

**Fig 12:**
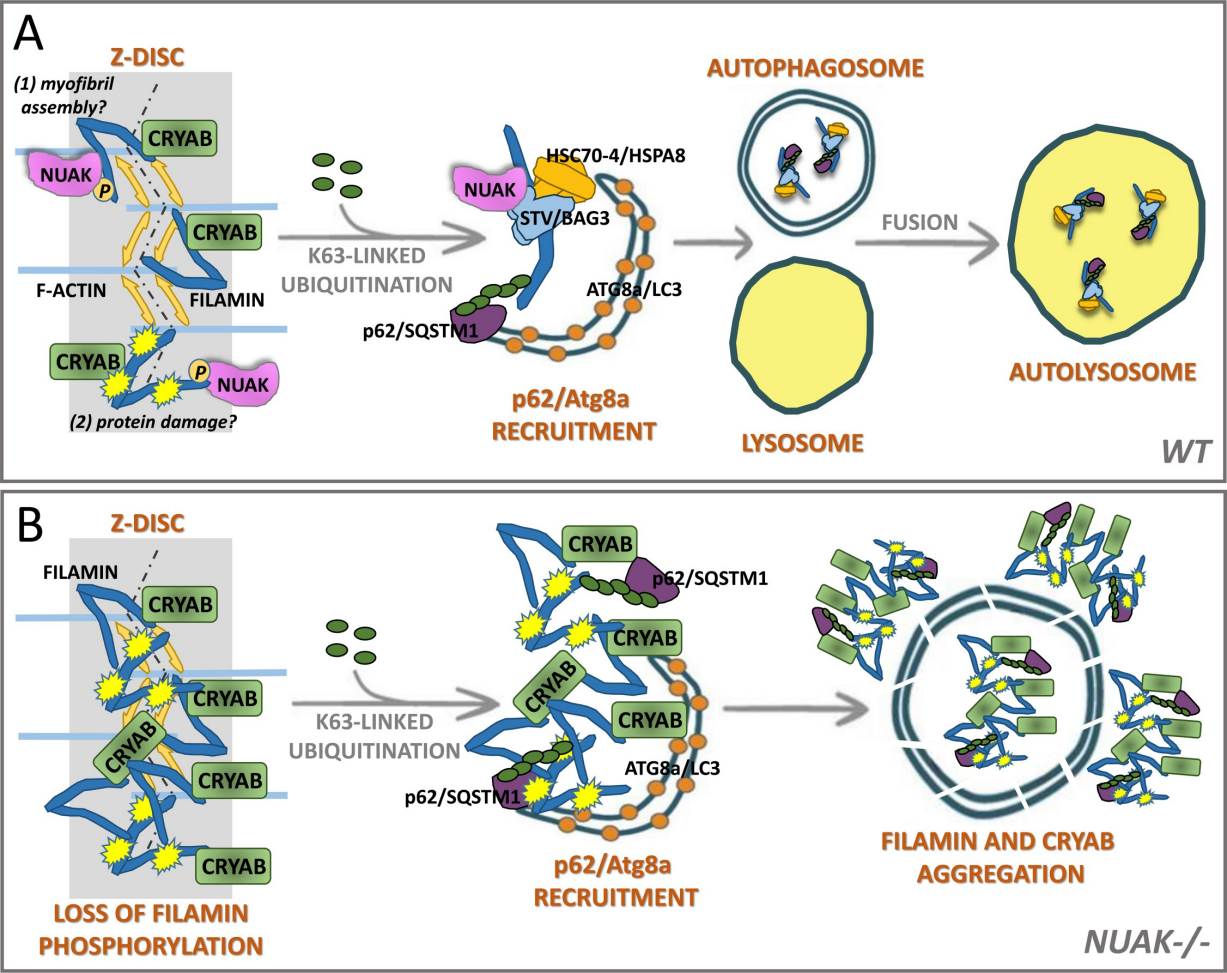
Model for NUAK function. (A) Autophagic protein clearance of Fil in *WT* muscle tissue. NUAK phosphorylates Fil either for incorporation into the Z-disc (1) and/or upon protein damage due to folding and unfolding of the protein (2). CryAB is normally bound to Fil at the Z-disc and the Stv-Hsc70-4 protein complex detects damaged Fil protein. The addition of K63-linked polyubiquitin chains on Fil recruits p62/SQSTM1 and Atg8a for autophagosome biogenesis. Autophagosomes fuse with lysosomes to form the autolyosome for eventual protein degradation. (B) Fil phosphorylation is lost in *NUAK-/-*. Unincorporated and/or damaged Fil protein at the Z-disc bound to CryAB. PolyUb, p62, and Atg8a proteins are still recruited to Fil-CryAB complexes. The absence of lysosomes in regions of protein accumulation prevents autophagic protein clearance.

We propose a model for NUAK that incorporates our new findings with existing roles for BAG3 (**Fig 12A**). Fil and CryAB are physically associated at the Z-disc in *Drosophila* larval muscle [39]. The phosphorylation of Fil by NUAK may control the incorporation of Fil into the Z-disc during myofibril assembly and/or may be required for the disposal of damaged Fil protein. BAG3 and chaperones such as Hsc70/HSPA8 are thought to monitor the MSR of Fil to detect force-induced damage and to promote the addition of K63-linked polyUb chains [6, 7, 16, 47]. Recruitment of the ubiquitin autophagic adapter p62/SQSTM1 induces autophagosome initiation through the accumulation of Atg8a. Eventual fusion of these autophagosomes with lysosomes promotes protein client complex destruction.

Upon loss of NUAK (**Fig 12B**), excess Fil protein that fails to be incorporated into the Z-disc and/or is damaged due to tension-induced muscle contraction begins to accumulate near the Z-disc. The presence of CryAB in Fil-like aggregates may be due to the normal association of CryAB with Fil at the Z-disc, either to monitor Fil protein damage, or to prevent protein aggregation [65–67]. It is interesting that while both Fil and CryAB contain actin-binding domains [39], these associations are lost in *NUAK-/-* muscle tissue as F-actin is displaced from regions of Fil-CryAB accumulation. At this point we cannot determine if NUAK preferentially binds to the short (~90kD) and/or long (~240 kD) Fil isoforms since the mapped interaction domains (Ig domains 15–18) are present in both isoforms.

In the initial stages of aggregate formation, nearly all Fil puncta are decorated with Ub. We hypothesize that the observed decrease in Ub-Fil colocalization in large regions of aggregate formation may be due to intrinsic properties of aggregation-prone proteins whereby protein misfolding triggers aggregation of Fil with itself and other proteins [68]. The accumulation of p62 and circular structures that stain positive for Atg8a in regions of Fil accumulation demonstrate that the autophagosome machinery is recruited to BAG3-client complexes. The absence of lysosomes in these aggregate regions suggest that either fusion and/or transport to sites of degradation are compromised.

CASA-mediated autophagy via the BAG3-client complex includes Hsc70-4/HSPA8, HSPB8, and the E3 ligase CHIP/STUB1, the latter of which ubiquitinates Fil for the subsequent recruitment of p62 to initiate autophagosome formation [11]. However, fibroblasts deficient for CHIP are not defective in autophagy and mice or flies lacking CHIP/STUB1 are viable [69, 70]. A failure to enhance protein aggregation defects upon *CHIP RNAi* knockdown in our sensitized *NUAK+/-* or *stv+/-* backgrounds suggests that additional Ub ligases cooperate with the Stv/BAG3 complex to remove damaged proteins (**S6 Fig**). Future studies will also determine which *Drosophila* protein is the equivalent of HSPB8 since we did not observe genetic interactions with putative *CG14207* or *Hsp67Bc RNAi* lines (**S6 Fig**). This negative data does not rule out the possibility that protein levels are not reduced enough to see phenotypes upon RNAi induction or possible functional redundancy exists between CG14207 and Hsp67Bc.

### Connections to protein aggregation disease

An interesting hallmark of protein aggregate diseases is the accumulation of specific proteins in affected cells or tissues. Thus, proteins susceptible to aggregation in vivo may possess specific structural characteristics or shared biological functions. This latter feature is evident in a group of protein aggregate diseases termed myofibrillar myopathies (MFM). Laser microdissection of aggregates from normal or affected muscles reveal specificity in the types of proteins that accumulate in patients afflicted with MFMs [71–73]. Common proteins present in these aggregates include Filamin C (FILC), αB-crystallin (CRYAB), BAG3, and Desmin (DES), among others. The inability of MFM patients to clear these aggregates results in myofibrillar degeneration and a decline in muscle function [74–80]. Interestingly, mutations in *Drosophila NUAK* phenocopy both structural and functional deficits observed in MFM patients, including Fil and CryAB accumulation, muscle degeneration, and locomotor defects. The discovery of cellular degeneration and protein aggregation in muscle tissue upon loss of the single fly NUAK ortholog highlights the power of *Drosophila* as a model. Future studies will focus on identifying kinase targets of NUAK and defining additional proteins that function in *NUAK* and *stv-*mediated autophagy for the eventual development of therapeutic targets to treat MFMs and other protein aggregate diseases.

## Materials and methods

### *Drosophila* stocks and growth conditions

#### Stocks

*Drosophila* stocks were obtained from the Bloomington (BL) *Drosophila* Stock Center (BDSC), the Vienna *Drosophila* Resource Center (VDRC), or the Kyoto *Drosophila* Genetic Resource Center (DGRC). The *NUAK* ^*l(3)17289*^ allele was isolated in an EMS screen [36] and analyzed over the deficiency stock *Df(3R)BSC479* (BL24983). Other mutant alleles used in experiments were *stv*^*1*^/*TM6*, *Tb* (a gift from Jörg Höhfeld) [11] and *stv*^*00543*^*/TM3*, *Sb* (BL11501). The following Gal4 lines were used to direct tissue-specific expression: *da*-Gal4 (originally BL37291 outcrossed ten times to *w*^*1118*^ to remove background lethal mutations), *elav*-Gal4 (BL458), *mef2*-Gal4 (BL27390), and *24B*-Gal4 (BL1767). RNAi lines used to knockdown transcript levels: UAS-*NUAK RNAi #1 (TRiP*.*JF02162*, BL31885), UAS-*NUAK RNAi #2* (*TRiP*.*GL00066*, BL35194), UAS-*stv RNAi #1* (*TRiP*.*HMJ02221*, BL42564), UAS-*stv RNAi #2* (*GD10796*, VDRC34408), UAS-*Hsc70-4 RNAi* (*TRiP*.*HMJ21529*, BL54810), and UAS-*Atg8a RNAi* (*GD4654*, VDRC43097). RNAi lines used to knockdown transcript levels in Supplemental Figures: UAS-*CHIP RNAi #1* (*KK108451*, VDRC107447), UAS-*CHIP RNAi #2* (*GD10538*, VDRC34124), UAS-*CG14207 RNAi* (*TRiP*.*HMC05590*, BL64571), UAS-*Hsp67Bc RNAi #1* (*TRiP*.*HMS02440*, BL42607), UAS-*Hsp67Bc RNAi #2* (*KK103547*, VDRC103974), and UAS-*cher RNAi* (*KK107518*, VDRC107451). Fly stocks containing GFP fusion proteins were UAS-*Atg8a-GFP* (BL51656) and Lamp1-GFP (CPTI001775, DGRC115240). UAS-stv-V5 and UAS-CG14207-V5 were a gift from Harm Kampinga [81]. Mutant alleles and lethal transgenes were maintained over the appropriate balancer chromosome: Cyo, Tb (II), or TM6, Tb (III). Homozygous mutant larvae were chosen by selection against the Tb marker.

#### Rearing conditions

Flies were raised on standard cornmeal medium at 25°C unless otherwise specified. *w*^*1118*^ was used as the *WT* control. All temperature-dependent crosses to analyze GAL4/UAS expression were performed at 29°C unless specified in the Figure legends. For starvation assay, larvae were removed from normal food at ~70 AEL and transferred to agar plates with a moist Kimwipe to prevent desiccation. Larvae that survived were dissected about 4 days later and stained with phalloidin.

### Mapping and sequencing of *NUAK* mutants

#### Mapping

Pupal lethal lines isolated from a previously described EMS screen [36] that exhibited elongated pupal cases were crossed to third chromosome deficiency stocks (n = 177) obtained from the BDSC. The progeny of each cross were screened for pupal lethality and extended pupal case morphology. The *l(3)17289* allele failed to complement *Df(3R)ED5474* (BL9082). Verification with additional overlapping Df stocks narrowed down the cytological region to 86A3 to 86B1.

#### Sequencing

Homozygous mutant *l(3)17289* L3 individuals were washed with 0.7% NaCl/0.04% Triton X-100 and thoroughly rinsed with ultrapure water. After homogenization, total RNA was extracted and purified using the RNeasy mini kit (Qiagen, Germantown, MD). Synthesis of cDNA was performed using the qScript XLT cDNA SuperMix kit (Quanta Biosciences, Beverly, MA). RT-PCR was performed using Promega GoTaq Flexi (Madison, WI) with NUAK_seq_forward 5’-TCATCGAACCGCAAGCTAC and NUAK_seq_reverse 5’- GTCCTCCTCGTTGGAGCTTT. Sanger sequencing was performed by GeneWiz using the following primer: 5’- GCTGCAGAGGGACCTACG.

### Mutagenesis and the creation of transgenic flies

Clone FI03914 corresponding to *NUAK-RD* was obtained from the *Drosophila* Genomics Resource Center (DGRC). The entirety of this *NUAK* open reading frame (ORF) was PCR amplified with the forward primer 5’-CACCATGGTGATAAGCAAACCCGATGG and the reverse primer 5’-CTACTGATCTAGGTATTTACTCTTTATTC. This fragment was inserted into the Gateway pENTR/D-TOPO vector (Invitrogen) and recombined into the pTW destination plasmid (DGRC) using standard procedures to generate UAS-*NUAK*. pENTR/D-TOPO_NUAK was used as a template to introduce the K99R and E197K mutations using the QuikChange II XL Site-directed Mutagenesis kit (Agilent Technologies, Santa Clara, CA). Primer sequences used were: NUAK_K99R forward 5’-TGCACTTCTTGATGGTTCTGATAGCCACCTCCTGG; NUAK_K99R reverse 5’-CCAGGAGGTGGCTATCAGAACCATCAAGAAGTGC; NUAK_E197K forward 5’- CGCGATCTCAAGCTGAAGAACATCCTGCTGG; NUAK_E197K reverse 5’- CCAGCAGGATGTTCTTCAGCTTGAGATCGCG. These mutagenized sequences were put into the pTW destination plasmid to generate UAS_NUAK_K99R and UAS_NUAK_E197K. All constructs were sequence verified and injected by Genetic Services, Inc. for the creation of transgenic flies.

### Immunostaining

Wandering L3 larvae were dissected to isolate muscle fillets and fixed in 4% formaldehyde as described [32, 34, 82]. Tissues were stained with the following primary antibodies: mouse anti-TM (1:50, Babraham Institute, Cambridge, UK), mouse anti-MHC (1:500, Susan Abmayr) [83], rabbit anti-Mlp84B (1:50, Kathleen Clark) [33], rabbit anti-Fil (1:300, Lynn Cooley) [84], rat anti-CryAB (1:400, Teresa Jagla) [39], mouse anti-Ub (1:300, Enzo Life Sciences, Farmingdale, NY), and rabbit anti-ref(2)p (1:200, Abcam, Cambridge, MA), anti-Atg8a (1:200, Millipore, Burlington, MA), and anti-Perl (1:500, Stephan Baumgartner) ([85], 1:2000). Amino acids 611–838 of Cher_PB were used as an antigen to immunize rabbits (Bosterbio, Pleasanton, CA). This antibody was used at 1:200 for immunostaining (**S9 Fig**). Fluorescence was detected using the following secondary antibodies: Alexa Flour anti-mouse 488, Alexa Flour anti-rabbit 488, or Alexa Flour anti-rat 488 (1:400, Molecular Probes, Eugene, OR). F-actin was labeled with phalloidin 488, 594, or 647 (1:400, Molecular Probes, Eugene, OR). Images were captured using a Zeiss 700 confocal microscope. Image processing and analysis was performed using a combination of Zen Black (Zeiss), ImageJ (NIH), and Adobe Photoshop. All images taken at 4, 10x, or 20x are displayed as maximum intensity projections. Data acquisition at increased magnifications (40x or 63x) are presented a single plane confocal micrographs.

### Transmission electron microscopy

*Drosophila* L3 larvae were filleted and fixed overnight in 1x Trump’s fixative (4% formaldehyde/1% glutaraldehyde in phosphate buffer) as in [86]. Fillets were processed with osmium tetroxide and put through a graded alcohol dehydration series before embedding in Spurr resin. Ultrathin sections of the dissected fillets were taken in a parasagittal orientation starting at the dorsal edges of muscle hemisegments using uranyl acetate and lead citrate for contrast. Samples were observed and imaged with a FEI Tecnai 12 Bio-spirit Transmission electron microscope in the Nanotechnology Innovation Center of Kansas State (NICKS). Images were prepared using the Gatan Microscopy Suite software.

### Phenotypic quantification & statistical analysis

#### Muscle defect quantification

Six muscles within each complete thoracic hemisegment (LL1, LO1, VL1-4) were used for each type of quantification. (1) Percent muscle defects were calculated by dividing the number of abnormal muscles (regions lacking F-actin as a proxy for protein aggregation) by the total number of muscles counted in each genotype. These percentages were compiled in GraphPad 6.0 and graphically represented as dot plots. (2) A similar type of calculation (% of each type of defect/total muscles counted per genotype) was used to classify *NUAK-/-* phenotypes. (3) Muscle severity was quantitated for *Atg8a* enhancement experiments. Mild defects included long, thin regions of empty space between adjacent myofibrils or a single F-actin(-) region per muscle. Severe defects included >2 regions lacking F-actin per muscle. N≥20 for each genotype

#### Muscle length and sarcomere number determination

Muscle fillets at the indicated stages were stained with phalloidin. The line function in Image J was used to draw a straight line across the length of the muscle. Muscle length was determined using the measure function. The number of sarcomeres was counted after using the plot function that measures peak corresponding to the Z-disc in each sarcomere.

#### Pupal case axial ratio determination

Pupa of the appropriate genotype were removed from vials, oriented dorsal side up, and attached to slides using a small drop of nail polish. Images were taken with a Leica M165 FC Stereomicroscope. Length and width measurements for each pupae were performed in ImageJ using the line and measure functions. Values were put into an Excel spreadsheet and the axial ratio (length/width) was calculated for each individual. The raw data was imported into Graphpad Prism 6.0 and graphed as a box and whiskers plot. N≥20 for each genotype.

#### Locomotion analysis

Larval locomotion studies were performed on apple juice agar plates as described [87]. N≥20 for each genotype. Note that all control larvae (*WT*, *mef2>+*, and *mef2>GFP RNAi*) appear to be biphasic, where a cohort moves fast and a similar cohort moves slower. Statistical analysis by One-Way ANOVA followed by the Kruskal-Wallis test does not show a statistical difference between these genotypes.

#### Rescue analysis

Defective muscles for each genotype were quantitated as described above using method #1. The percentage of muscle defects was subtracted from 100% and graphed as shown. Experiments were performed at 18°C. N≥20 for each genotype.

#### Puncta quantification

The number of Ub(+) or p62(+) puncta in a 20 um^2^ area was manually counted within muscles of the indicated genotypes in regions that lacked phalloidin staining. Analysis was performed in ImageJ. N≥20 for each genotype.

#### Statistical analysis

Statistical analyses were performed in GraphPad 6.0. The unpaired student t-test was used to evaluate the significance between two groups. All other data sets that compared three or more unmatched groups were subjected to one-way ANOVA analysis. Data points in each graph were first analyzed for Gaussian distribution sampling. Data sets that conformed to these parameters used the Mann-Whitney test. The nonparametric Kruskal-Wallis test was used to compare three or more unmatched groups that did not conform to a Gaussian distribution. Significance values are indicated in each figure legend and in **S1 Table**.

### Quantitative RT-PCR

Transcript levels were assessed using quantitative PCR (qPCR). Total RNA was collected from three wandering L3 larvae in triplicate using the RNeasy Mini Kit (Qiagen, Hilden, Germany). Synthesis of cDNA from 150 ng RNA (*NUAK* and *stv RNAi*) or 300 ng RNA (*Hsc70-4* and *Atg8a RNAi*)was performed using the qScript XLT cDNA SuperMix kit (Quanta Biosciences, Beverly, MA). Dilutions of cDNA were optimized according to each primer set (1:10 to 1:100) and combined with PowerUp SYBR Green Master Mix (ThermoFisher, Waltham, MA). The following primers were used: *rp49* forward 5’-GCCCAAGGGTATCGACAACA, reverse 5’-GCGCTTGTTCGATCCGTAAC; *NUAK* forward 5-CAGTTCCAACACAACCACGC, reverse 5’-GGATGATAAACTCCCGCGGA; *stv* forward 5’-GTTCCTCCAAATCAGCAGCA, reverse 5’-CAAAGTGTGAGTCGCCGAAG; *Hsc70-4* forward 5’-TGG GCA AGA CTG TGA CCA AC, reverse 5’- TCC AGA CCG TAA GCG ATA GCA; *Atg8a* forward 5’-GGATGCCCTCTTCTTCTTTGTG, reverse 3’-CGGAGTAGGCAATGTACAGGA; *cher* forward 5’-GCCCTTCCAGCCACTAATAGT, reverse 5’-GCTGCCCACCTTGCTCATAT; *l(2)efl/CryAB* forward 5’-TTCCACCCTCAACATCGACA, reverse 5’-CATGCTTTCCCTCCACGATG; *ref(2)p/p62* forward 5’- GCCCTCCCAGAATTACACCA, reverse 5’- GTTGGCCGAAGAACCCTCT. All primers were synthesized at Integrated DNA Technologies (IDT, Stokie, IL). Quantitative transcript levels were obtained using the 2-ΔΔCt method and graphed as Mean +/- SEM using GraphPad 6.0.

### Gel electrophoresis and Western blotting

#### 1D

Whole larvae of the appropriate genotype were placed into SDS sample buffer, boiled at 95°C for 3 min, homogenized to break up aggregates, boiled for an additional 10 min at 95°C, and centrifuged at 20,000xg for 10 min to pellet debris. The resulting protein samples were separated by sodium dodecyl sulfate polyacrylamide gel electrophoresis (SDS-PAGE), transferred to polyvinyl difluoride (PVDF) membranes (Pierce Biotechnology, Inc., Waltham, MA), and probed with rabbit anti-ref(2)p ab178440 (1:2000, Abcam, Cambridge, UK) and mouse anti-ATP5α (1:1000, Abcam, Cambridge, United Kingdom) as a loading control. Horseradish Peroxidase (HRP) conjugated secondary antibodies (1:5000–1:10000, GE Healthcare, Chicago, IL) were developed using the Prometheus ProSignal Pico detection system (Genesee Scientific, San Diego, CA) and imaged with the FluorChem M system (Protein Simple, San Jose, CA). Quantification of Western blot protein levels was performed using standard densiometric analysis functions in ImageJ.

#### 2D

Five dissected *WT* or *NUAK-/-* larvae were homogenized in 50mM Tris + 1% SDS and centrifuged at 21,130 xg for 15 minutes. 10μL of the samples were then added to 115μL of 2D sample buffer (8M urea, 2% CHAPS, 0.05M DTT, 1X Biolyte 3–10, and 0.001% bromophenol blue). The samples were transferred to separate lanes of a BioRad Protean IEF focusing tray along with BioRad ReadyStrip IPG strips, pH 5–8, 7cm (BioRad Laboratories, Hercules, CA) and covered with mineral oil. Using a BioRad Protean IEF cell, the strips were passively rehydrated (20°C for 12 hrs) and then focused (4000V, Rapid, 15000 Vhrs). After focusing, the strips were equilibrated in DTT containing buffer (6M urea, 0.375M Tris-HCL pH = 8.8, 2% SDS, 20% glycerol, and 2% (w/v) DTT) for 15 minutes and then iodoacetamide containing buffer (6M urea, 0.375M Tris-HCL pH = 8.8, 2% SDS, 20% glycerol, and 2.5% (w/v) iodoacetamide) for 15 minutes and briefly rinsed with standard SDS-PAGE running buffer. 2^nd^ dimension separation was achieved using 7% SDS-PAGE gels. Proteins were then transferred to PVDF membranes (Millipore, Burlington, MA) using BioRad’s Trans-Blot Turbo Transfer System (BioRad Laboratories, Hercules, CA). Membranes were blocked for one hour in Prometheus OneBlock Western-CL Blocking Buffer (Genesee Scientific, Can Diego, CA), incubated overnight in rabbit anti-Fil (1:5000, see **S6 Fig**) in the same blocking buffer, washed, blocked for 30 minutes, incubated for 2 hrs at room temperature with 1:10,000 ECL rabbit HRP secondary (GE Healthcare Chicago, IL) in the blocking buffer, washed, and developed with Prometheus ProSignal Pico substrate (Genesee Scientific, Can Diego, CA). Images were obtain using a Protein Simple FluorChem M system.

#### Soluble/Insoluble fractionation

Muscle carcasses from ten dissected *WT* or *NUAK-/-* larvae were homogenized in 100μL ice cold RIPA buffer (50mM Tris pH = 8, 150mM NaCl, 2mM EDTA, 1% tritonX, 0.1% SDS, 1%NaDexoxycholate, and Halt Protease Inhibitors Cocktail (ThermoFisher, Waltham, MA). The homogenate was centrifuged at 21,130 xg for 10 minutes at 4°C. The supernatant (RIPA buffer soluble sample) was transferred to a clean tube, 10μL were removed for protein determination by BCA assay, and 100μL SDS-PAGE sample buffer (62.5mM Tris pH = 6.8, 25% glycerol, 2% SDS, 0.01% Bromophenol Blue, 5% B-mercaptoethanol) was added. The pellet from the RIPA buffer homogenization was washed twice with 500μL phosphate buffered saline containing Halt Protease Inhibitor Cocktail (ThermoFisher, Waltham, MA). 100μL of room temperature Urea buffer (9M Urea, 50mM Tris pH = 8, 1% CHAPS, and halt protease inhibitors) was added and the pellet was re-homogenized. The resulting mixture was centrifuged at 21,130 xg for 10 minutes at 4°C. The supernatant (Urea buffer soluble sample) was transferred to a clean tube, 10μL were removed for a BCA assay, and 100μL SDS-PAGE sample buffer was added. The remaining pellet was washed once with 1mL phosphate buffered saline containing Halt Protease Inhibitors and then re-homogenized in 100μL SDS-PAGE sample buffer (SDS buffer soluble sample). For the RIPA and Urea soluble samples, protein concentration was determined using Pierce BCA Protein Assay kit (ThermoFisher, Waltham, MA). All three samples were boiled 5–10 minutes and centrifuged before loading to a SDS-PAGE gel. For RIPA and Urea soluble samples, 10μg of total protein was loaded; for SDS buffer samples, 10–15μL was loaded. Western blotting was performed as described for 1D and 2Dgels using rabbit anti-Fil (1:5000) and rabbit anti-Ubi-K63 (1:1000, Enzo Life Sciences, Farmingdale, NY).

### Yeast 2-hybrid screen and verification

#### Screen

Y2H screens were performed by Hybrigenics Services. The coding sequence of the full-length *Drosophila* NUAK/CG43143 (isoform D) was PCR-amplified and cloned in frame with the Gal4 DNA binding domain (DBD) into plasmid pB66 as a C-terminal fusion to Gal4 (Gal4-bait fusion) (Fromont-Racine et al., 1997). *Drosophila* Stv/CG10745 was also PCR-amplified and cloned in frame with the DBD as a C-terminal fusion to Gal4 into plasmid pB35. These NUAK-DBD or Stv-DBD bait proteins were independently used to screen a *Drosophila* L3 larval library at 2.0 mM or 10.0 mM 3AT respectively. 57.5 and 52.9 million interactions were analyzed for NUAK and STV, respectively. All interacting prey fragments were sequenced.

#### Verification

For 1 x 1 interaction assays with NUAK, the prey fragment corresponding to amino acids 322–516 of Stv were cloned in frame with the Gal4 activation domain (AD) into plasmid pP6, derived from the original pGADGH plasmid (Bartel et al. 1993). Bait and prey constructs were transformed in the yeast haploid cells CG1945 (mata) and YHGX13 (Y187ade2-101::loxP-kanMX-loxP, matα), respectively. The diploid yeast cells were obtained using a mating protocol with both yeast strains (Fromont-Racine et al., 1997). These assays are based on the HIS3 reporter gene (growth assay without histidine). As negative controls, the bait plasmid was tested in the presence of empty prey vector (pP7) and all prey plasmids were tested with the empty bait vector (pB66). Controls and interactions were tested in the form of streaks of three independent yeast clones for each control and interaction on DO-2 and DO-3 selective media. The DO-2 selective medium lacking tryptophan and leucine was used as a growth control and to verify the presence of the bait and prey plasmids. The DO-3 selective medium without tryptophan, leucine, and histidine selects for the interaction between bait and prey.

## Supporting information

S1 Table Raw data and statistics summary. Graph type, n values, statistical tests and p-values for all quantitative analysis.
(DOCX)

S1 Fig The *l(3)17289* mutation maps to *CG41343*. (A) Deficiency mapping narrowed down the *l(3) 17289* mutation to Df(3R)BSC479. This deficiency removes ten genes. (B) RT-PCR and Sanger sequencing of CG43143 revealed a C>T change that results in a stop codon.
(TIF)

S2 Fig Induction of *NUAK RNAi* causes muscle degeneration. (A-B’) L3 muscle fillets stained with phalloidin after knockdown of NUAK using two independent UAS-RNAi insertions (*UAS-NUAK* ^*RNAi #1*^ or *UAS-NUAK* ^*RNAi #2*^). (A-B’) 4x magnification (A,B) or 10x view of one hemisegment (A’,B’) shows thinner (carets) or detached (arrowhead) muscles. (A”,B”) 20x image of a representative VL3 muscle show areas lacking F-actin (white dotted outline). (C) qPCR verifies that *NUAK* transcript levels are reduced ~50% after induction of either *UAS-NUAK* ^*RNAi #1*^ or *UAS-NUAK* ^*RNAi #2*^ using the ubiquitous *daughterless (da)-*Gal4 driver. (D) Box and whisker plot depicting axial ratios of pupal length upon ubiquitous knockdown of *NUAK RNAi* with *da*-Gal4. (E,F) Whole mount L1 larval muscles visualized with MHC-GFP. Thinner (white carets) or altered muscle pattern (white arrowhead) is observed upon loss of NUAK. Mean +/- SEM (**, p<0.01; ***, p<0.005; ****, p<0.001).
(TIF)

S3 Fig Tissue degeneration occurs upon a decrease of NUAK in muscle, but not neurons. (A-D) L3 muscles within a single hemisegment stained with phalloidin. (A) Expression of the *mef2* driver alone shows no phenotype. (B) *mef2*-driven *NUAK RNAi* results in morphological muscle defects. (C,D) Neither the neuronal driver *C155* alone (C) or inducing *NUAK RNAi* (D) causes muscle phenotypes. (E) Scatter plot shows that muscle defects are only apparent upon NUAK RNAi induction in muscle, but not neuronal tissue. (F) Scatter plot of larval locomotor ability upon muscle (*mef2*) or neuronal (*C155*) RNAi knockdown of NUAK. Larvae were transferred from 25°C to 29°C after hatching. Mean +/- SEM (*, p<0.05; ****, p<0.001; n.s., not significant).
(TIF)

S4 Fig Fil does not accumulate in torn muscles. (A-B”) Intentionally torn L3 muscles stained for F-actin (green) do not accumulate Fil protein (purple, asterisk). (C,D’) *NUAK-/-* with regions devoid of F-actin (green, white arrow) have an intact basement membrane visualized by Perlecan (purple).
(TIF)

S5 Fig Two independent *stv RNAi* lines show muscle morphology defects. (A-B’) 10x or 20 x images of a single hemisegment of the L3 musculature stained with phalloidin (green). Expression of both *stv RNAi* insertions in muscle show regions that where F-actin is excluded (* in A,B; white dotted lines in A’,B’). (C) Both the *UAS-stv* ^*RNAi #1*^ or *UAS-stv*^*RNAi #2*^ *RNAi* lines effectively decrease *stv mRNA* levels as assayed by qPCR. Mean +/- SEM (**, p<0.01).
(TIF)

S6 Fig Genetic interactions with CASA pathway components. (A,B) One hemisegment of the L3 musculature stained with phalloidin. Defects caused by knockdown of *NUAK RNAi* (A) can be rescued upon re-introduction of *NUAK cDNA* in muscle tissue. (C) Bar graph showing NUAK rescue results. NUAK is capable of restoring muscle defects due to loss of NUAK, but not Stv, Hsc70-4, or Atg8a. (D,E) Scatter plots of genetic interactions with *NUAK* (D) or *stv* (E). Mean +/- SEM (*, p<0.05; ****, p<0.001).
(TIF)

S7 Fig *Hsc70-4* and *Atg8a* muscle phenotypes upon RNAi knockdown. (A,C) F-actin labeled muscles in two hemisegments of the L3 musculature. (A) Nearly all muscles of the genotype *mef2>Hsc70-4* show abnormal morphology (*). (A’) Regions devoid of F-actin are outlined (white dashed lines). (B) Bar graph shows a decrease in *Hsc70-4 mRNA* levels driven with *mef2*-Gal4. (C) RNAi knockdown of *Atg8a mRNA* affects muscles to a lesser extent. (C’) The predominant phenotype is the presence of dark regions, indicative of protein aggregation. (D) Bar graph illustrating that the UAS-*Atg8a RNAi* insertion effectively reduces transcript levels. Mean +/- SEM (*, p<0.05; **, p<0.01).
(TIF)

S8 Fig *p62* transcripts are increased in *NUAK-/-* and *stv-/-*. Bar graphs showing the indicated transcripts in *NUAK-/-* or *stv-/-*. *cher*, *CryAB*, *Hsc70-4*, and *Atg8a mRNA* levels are not altered upon loss of NUAK or Stv (left panel). *stv* transcript levels are mildly increased in *NUAK* mutants, but *NUAK* transcripts do not change upon loss of Stv (middle panel). *p62 mRNA* levels are much higher in both *NUAK* and *stv* mutants (right panel). Mean +/- SEM (*, p<0.05; **, p<0.01; n.s., not significant).
(TIF)

S9 Fig Characterization of Fil antisera. (A-B”) Anti-Fil (green) and F-actin (purple) staining of L3 muscles VL3 and VL4 in control (*mef2>+)* or upon a decrease in *cher mRNA* levels (*mef2>cher*^*RNAi*^). The striated pattern of Fil immunostaining (A,A”) is blunted upon targeted induction of *cher RNAi* in muscle tissue (B, B”). (C) Western blot showing a decrease in the 90 kD form of Fil after knockdown of *cher* transcripts.
(TIF)

## References

[1] Jia B, Wu Y, Zhou Y. 14-3-3 and aggresome formation: implications in neurodegenerative diseases. Prion. 2014;8(2). Epub 2014/02/18. 10.4161/pri.28123 24549097 PMC4189886

[2] Takalo M, Salminen A, Soininen H, Hiltunen M, Haapasalo A. Protein aggregation and degradation mechanisms in neurodegenerative diseases. Am J Neurodegener Dis. 2013;2(1):1–14. Epub 2013/03/08. 23516262 PMC3601466

[3] Shamsi TN, Athar T, Parveen R, Fatima S. A review on protein misfolding, aggregation and strategies to prevent related ailments. Int J Biol Macromol. 2017;105(Pt 1):993–1000. Epub 2017/07/23. 10.1016/j.ijbiomac.2017.07.116 . 28743576

[4] Klaips CL, Jayaraj GG, Hartl FU. Pathways of cellular proteostasis in aging and disease. J Cell Biol. 2018;217(1):51–63. Epub 2017/11/10. 10.1083/jcb.201709072 29127110 PMC5748993

[5] Kaushik S, Cuervo AM. Chaperones in autophagy. Pharmacol Res. 2012;66(6):484–93. Epub 2012/10/08. 10.1016/j.phrs.2012.10.002 23059540 PMC3502706

[6] Klimek C, Kathage B, Wördehoff J, Höhfeld J. BAG3-mediated proteostasis at a glance. J Cell Sci. 2017;130(17):2781–8. Epub 2017/08/14. 10.1242/jcs.203679 . 28808089

[7] Stürner E, Behl C. The Role of the Multifunctional BAG3 Protein in Cellular Protein Quality Control and in Disease. Front Mol Neurosci. 2017;10:177 Epub 2017/06/21. 10.3389/fnmol.2017.00177 28680391 PMC5478690

[8] Kettern N, Dreiseidler M, Tawo R, Höhfeld J. Chaperone-assisted degradation: multiple paths to destruction. Biol Chem. 2010;391(5):481–9. 10.1515/BC.2010.058 . 20302520

[9] Ulbricht A, Höhfeld J. Tension-induced autophagy: may the chaperone be with you. Autophagy. 2013;9(6):920–2. Epub 2013/03/21. 10.4161/auto.24213 23518596 PMC3672301

[10] Ulbricht A, Arndt V, Höhfeld J. Chaperone-assisted proteostasis is essential for mechanotransduction in mammalian cells. Commun Integr Biol. 2013;6(4):e24925 Epub 2013/06/11. 10.4161/cib.24925 23986815 PMC3737759

[11] Arndt V, Dick N, Tawo R, Dreiseidler M, Wenzel D, Hesse M, et al Chaperone-assisted selective autophagy is essential for muscle maintenance. Curr Biol. 2010;20(2):143–8. Epub 2010/01/07. 10.1016/j.cub.2009.11.022 . 20060297

[12] Ulbricht A, Eppler FJ, Tapia VE, van der Ven PF, Hampe N, Hersch N, et al Cellular mechanotransduction relies on tension-induced and chaperone-assisted autophagy. Curr Biol. 2013;23(5):430–5. Epub 2013/02/21. 10.1016/j.cub.2013.01.064 . 23434281

[13] Ulbricht A, Gehlert S, Leciejewski B, Schiffer T, Bloch W, Höhfeld J. Induction and adaptation of chaperone-assisted selective autophagy CASA in response to resistance exercise in human skeletal muscle. Autophagy. 2015;11(3):538–46. 10.1080/15548627.2015.1017186 25714469 PMC4502687

[14] Fürst DO, Goldfarb LG, Kley RA, Vorgerd M, Olivé M, van der Ven PF. Filamin C-related myopathies: pathology and mechanisms. Acta Neuropathol. 2013;125(1):33–46. Epub 2012/10/30. 10.1007/s00401-012-1054-9 23109048 PMC5127197

[15] Razinia Z, Mäkelä T, Ylänne J, Calderwood DA. Filamins in mechanosensing and signaling. Annu Rev Biophys. 2012;41:227–46. Epub 2012/02/23. 10.1146/annurev-biophys-050511-102252 22404683 PMC5508560

[16] Gamerdinger M, Kaya AM, Wolfrum U, Clement AM, Behl C. BAG3 mediates chaperone-based aggresome-targeting and selective autophagy of misfolded proteins. EMBO Rep. 2011;12(2):149–56. Epub 2011/01/21. 10.1038/embor.2010.203 21252941 PMC3049430

[17] Shaid S, Brandts CH, Serve H, Dikic I. Ubiquitination and selective autophagy. Cell Death Differ. 2013;20(1):21–30. Epub 2012/06/22. 10.1038/cdd.2012.72 22722335 PMC3524631

[18] Tanaka Y, Guhde G, Suter A, Eskelinen EL, Hartmann D, Lüllmann-Rauch R, et al Accumulation of autophagic vacuoles and cardiomyopathy in LAMP-2-deficient mice. Nature. 2000;406(6798):902–6. 10.1038/35022595 . 10972293

[19] Sun X, Gao L, Chien HY, Li WC, Zhao J. The regulation and function of the NUAK family. J Mol Endocrinol. 2013;51(2):R15–22. 10.1530/JME-13-0063 . 23873311

[20] Hawley SA, Boudeau J, Reid JL, Mustard KJ, Udd L, Mäkelä TP, et al Complexes between the LKB1 tumor suppressor, STRAD alpha/beta and MO25 alpha/beta are upstream kinases in the AMP-activated protein kinase cascade. J Biol. 2003;2(4):28 Epub 2003/09/24. 10.1186/1475-4924-2-28 14511394 PMC333410

[21] Lefebvre DL, Rosen CF. Regulation of SNARK activity in response to cellular stresses. Biochim Biophys Acta. 2005;1724(1–2):71–85. Epub 2005/04/08. 10.1016/j.bbagen.2005.03.015 . 15893879

[22] Koh HJ, Toyoda T, Fujii N, Jung MM, Rathod A, Middelbeek RJ, et al Sucrose nonfermenting AMPK-related kinase (SNARK) mediates contraction-stimulated glucose transport in mouse skeletal muscle. Proc Natl Acad Sci U S A. 2010;107(35):15541–6. 10.1073/pnas.1008131107 20713714 PMC2932588

[23] Lessard SJ, Rivas DA, So K, Koh HJ, Queiroz AL, Hirshman MF, et al The AMPK-related kinase SNARK regulates muscle mass and myocyte survival. J Clin Invest. 2016;126(2):560–70. 10.1172/JCI79197 26690705 PMC4731174

[24] Fisher JS, Ju JS, Oppelt PJ, Smith JL, Suzuki A, Esumi H. Muscle contractions, AICAR, and insulin cause phosphorylation of an AMPK-related kinase. Am J Physiol Endocrinol Metab. 2005;289(6):E986–92. Epub 2005/07/19. 10.1152/ajpendo.00335.2004 16030062 PMC1350986

[25] Hirano M, Kiyonari H, Inoue A, Furushima K, Murata T, Suda Y, et al A new serine/threonine protein kinase, Omphk1, essential to ventral body wall formation. Dev Dyn. 2006;235(8):2229–37. 10.1002/dvdy.20823 . 16715502

[26] Inazuka F, Sugiyama N, Tomita M, Abe T, Shioi G, Esumi H. Muscle-specific knock-out of NUAK family SNF1-like kinase 1 (NUAK1) prevents high fat diet-induced glucose intolerance. J Biol Chem. 2012;287(20):16379–89. 10.1074/jbc.M111.302687 22418434 PMC3351321

[27] Tsuchihara K, Ogura T, Fujioka R, Fujii S, Kuga W, Saito M, et al Susceptibility of Snark-deficient mice to azoxymethane-induced colorectal tumorigenesis and the formation of aberrant crypt foci. Cancer Sci. 2008;99(4):677–82. Epub 2007/02/27. 10.1111/j.1349-7006.2008.00734.x . 18307533 PMC11158890

[28] Hoppe PE, Chau J, Flanagan KA, Reedy AR, Schriefer LA. Caenorhabditis elegans unc-82 encodes a serine/threonine kinase important for myosin filament organization in muscle during growth. Genetics. 2010;184(1):79–90. 10.1534/genetics.109.110189 19901071 PMC2815932

[29] Schiller NR, Duchesneau CD, Lane LS, Reedy AR, Manzon ER, Hoppe PE. The Role of the UNC-82 Protein Kinase in Organizing Myosin Filaments in Striated Muscle of. Genetics. 2017;205(3):1195–213. Epub 2016/12/30. 10.1534/genetics.116.193029 28040740 PMC5340333

[30] Amin N, Khan A, St Johnston D, Tomlinson I, Martin S, Brenman J, et al LKB1 regulates polarity remodeling and adherens junction formation in the Drosophila eye. Proc Natl Acad Sci U S A. 2009;106(22):8941–6. 10.1073/pnas.0812469106 19443685 PMC2690039

[31] Couderc JL, Richard G, Vachias C, Mirouse V. Drosophila LKB1 is required for the assembly of the polarized actin structure that allows spermatid individualization. PLoS One. 2017;12(8):e0182279 Epub 2017/08/02. 10.1371/journal.pone.0182279 28767695 PMC5540607

[32] LaBeau-DiMenna EM, Clark KA, Bauman KD, Parker DS, Cripps RM, Geisbrecht ER. Thin, a Trim32 ortholog, is essential for myofibril stability and is required for the integrity of the costamere in Drosophila. Proc Natl Acad Sci U S A. 2012;109(44):17983–8. 10.1073/pnas.1208408109 23071324 PMC3497806

[33] Clark KA, Bland JM, Beckerle MC. The Drosophila muscle LIM protein, Mlp84B, cooperates with D-titin to maintain muscle structural integrity. J Cell Sci. 2007;120(Pt 12):2066–77. 10.1242/jcs.000695 . 17535853

[34] Green N, Odell N, Zych M, Clark C, Wang ZH, Biersmith B, et al A Common Suite of Coagulation Proteins Function in Drosophila Muscle Attachment. Genetics. 2016 Epub 2016/08/31. 10.1534/genetics.116.189787 27585844 PMC5105843

[35] Fogerty FJ, Fessler LI, Bunch TA, Yaron Y, Parker CG, Nelson RE, et al Tiggrin, a novel Drosophila extracellular matrix protein that functions as a ligand for Drosophila alpha PS2 beta PS integrins. Development. 1994;120(7):1747–58. . 7924982 10.1242/dev.120.7.1747

[36] Wang L, Evans J, Andrews HK, Beckstead RB, Thummel CS, Bashirullah A. A genetic screen identifies new regulators of steroid-triggered programmed cell death in Drosophila. Genetics. 2008;180(1):269–81. Epub 2008/08/30. 10.1534/genetics.108.092478 18757938 PMC2535680

[37] Bate M. The embryonic development of larval muscles in Drosophila. Development. 1990;110(3):791–804. . 2100994 10.1242/dev.110.3.791

[38] Tatum EL, Beadle GW. DEVELOPMENT OF EYE COLORS IN DROSOPHILA: SOME PROPERTIES OF THE HORMONES CONCERNED. J Gen Physiol. 1938;22(2):239–53. 10.1085/jgp.22.2.239 19873102 PMC2141983

[39] Wójtowicz I, Jabłońska J, Zmojdzian M, Taghli-Lamallem O, Renaud Y, Junion G, et al Drosophila small heat shock protein CryAB ensures structural integrity of developing muscles, and proper muscle and heart performance. Development. 2015;142(5):994–1005. 10.1242/dev.115352 . 25715399

[40] Zagórska A, Deak M, Campbell DG, Banerjee S, Hirano M, Aizawa S, et al New roles for the LKB1-NUAK pathway in controlling myosin phosphatase complexes and cell adhesion. Sci Signal. 2010;3(115):ra25 10.1126/scisignal.2000616 . 20354225

[41] Carrera AC, Alexandrov K, Roberts TM. The conserved lysine of the catalytic domain of protein kinases is actively involved in the phosphotransfer reaction and not required for anchoring ATP. Proc Natl Acad Sci U S A. 1993;90(2):442–6. 10.1073/pnas.90.2.442 8421674 PMC45679

[42] Reimann L, Wiese H, Leber Y, Schwäble AN, Fricke AL, Rohland A, et al Myofibrillar Z-discs Are a Protein Phosphorylation Hot Spot with Protein Kinase C (PKCα) Modulating Protein Dynamics. Mol Cell Proteomics. 2017;16(3):346–67. Epub 2016/12/27. 10.1074/mcp.M116.065425 28028127 PMC5340999

[43] Deshmukh A, Coffey VG, Zhong Z, Chibalin AV, Hawley JA, Zierath JR. Exercise-induced phosphorylation of the novel Akt substrates AS160 and filamin A in human skeletal muscle. Diabetes. 2006;55(6):1776–82. 10.2337/db05-1419 . 16731842

[44] Murray JT, Campbell DG, Peggie M, Mora A, Alfonso M, Cohen P. Identification of filamin C as a new physiological substrate of PKBalpha using KESTREL. Biochem J. 2004;384(Pt 3):489–94. 10.1042/BJ20041058 15461588 PMC1134134

[45] Brand AH, Perrimon N. Targeted gene expression as a means of altering cell fates and generating dominant phenotypes. Development. 1993;118(2):401–15. . 8223268 10.1242/dev.118.2.401

[46] Coulson M, Robert S, Saint R. Drosophila starvin encodes a tissue-specific BAG-domain protein required for larval food uptake. Genetics. 2005;171(4):1799–812. Epub 2005/09/02. 10.1534/genetics.105.043265 16143622 PMC1456105

[47] Rosati A, Graziano V, De Laurenzi V, Pascale M, Turco MC. BAG3: a multifaceted protein that regulates major cell pathways. Cell Death Dis. 2011;2:e141 Epub 2011/04/07. 10.1038/cddis.2011.24 21472004 PMC3122056

[48] Knezevic T, Myers VD, Gordon J, Tilley DG, Sharp TE, Wang J, et al BAG3: a new player in the heart failure paradigm. Heart Fail Rev. 2015;20(4):423–34. 10.1007/s10741-015-9487-6 25925243 PMC4463985

[49] Behl C. BAG3 and friends: co-chaperones in selective autophagy during aging and disease. Autophagy. 2011;7(7):795–8. 10.4161/auto.7.7.15844 . 21681022

[50] Kabbage M, Dickman MB. The BAG proteins: a ubiquitous family of chaperone regulators. Cell Mol Life Sci. 2008;65(9):1390–402. 10.1007/s00018-008-7535-2 . 18264803 PMC11131705

[51] Takayama S, Xie Z, Reed JC. An evolutionarily conserved family of Hsp70/Hsc70 molecular chaperone regulators. J Biol Chem. 1999;274(2):781–6. 10.1074/jbc.274.2.781 . 9873016

[52] Takayama S, Reed JC. Molecular chaperone targeting and regulation by BAG family proteins. Nat Cell Biol. 2001;3(10):E237–41. 10.1038/ncb1001-e237 . 11584289

[53] Lamark T, Johansen T. Aggrephagy: selective disposal of protein aggregates by macroautophagy. Int J Cell Biol. 2012;2012:736905 Epub 2012/03/22. 10.1155/2012/736905 22518139 PMC3320095

[54] Lim J, Yue Z. Neuronal aggregates: formation, clearance, and spreading. Dev Cell. 2015;32(4):491–501. 10.1016/j.devcel.2015.02.002 25710535 PMC4376477

[55] Lee YK, Lee JA. Role of the mammalian ATG8/LC3 family in autophagy: differential and compensatory roles in the spatiotemporal regulation of autophagy. BMB Rep. 2016;49(8):424–30. 10.5483/BMBRep.2016.49.8.081 27418283 PMC5070729

[56] Abdollahzadeh I, Schwarten M, Gensch T, Willbold D, Weiergräber OH. The Atg8 Family of Proteins-Modulating Shape and Functionality of Autophagic Membranes. Front Genet. 2017;8:109 Epub 2017/08/28. 10.3389/fgene.2017.00109 28894458 PMC5581321

[57] Erdi B, Nagy P, Zvara A, Varga A, Pircs K, Ménesi D, et al Loss of the starvation-induced gene Rack1 leads to glycogen deficiency and impaired autophagic responses in Drosophila. Autophagy. 2012;8(7):1124–35. Epub 2012/05/07. 10.4161/auto.20069 22562043 PMC3429548

[58] Chintapalli VR, Wang J, Dow JA. Using FlyAtlas to identify better Drosophila melanogaster models of human disease. Nat Genet. 2007;39(6):715–20. 10.1038/ng2049 . 17534367

[59] Vereshchagina N, Bennett D, Szöor B, Kirchner J, Gross S, Vissi E, et al The essential role of PP1beta in Drosophila is to regulate nonmuscle myosin. Mol Biol Cell. 2004;15(10):4395–405. Epub 2004/07/21. 10.1091/mbc.E04-02-0139 15269282 PMC519135

[60] Mizuno T, Tsutsui K, Nishida Y. Drosophila myosin phosphatase and its role in dorsal closure. Development. 2002;129(5):1215–23. . 11874917 10.1242/dev.129.5.1215

[61] Kathage B, Gehlert S, Ulbricht A, Lüdecke L, Tapia VE, Orfanos Z, et al The cochaperone BAG3 coordinates protein synthesis and autophagy under mechanical strain through spatial regulation of mTORC1. Biochim Biophys Acta. 2017;1864(1):62–75. Epub 2016/10/15. 10.1016/j.bbamcr.2016.10.007 . 27756573

[62] Modarres HP, Mofradt MR. Filamin: a structural and functional biomolecule with important roles in cell biology, signaling and mechanics. Mol Cell Biomech. 2014;11(1):39–65. . 25330623

[63] Puissant A, Fenouille N, Auberger P. When autophagy meets cancer through p62/SQSTM1. Am J Cancer Res. 2012;2(4):397–413. Epub 2012/06/28. 22860231 PMC3410580

[64] Klionsky DJ, Abdelmohsen K, Abe A, Abedin MJ, Abeliovich H, Acevedo Arozena A, et al Guidelines for the use and interpretation of assays for monitoring autophagy (3rd edition). Autophagy. 2016;12(1):1–222. 10.1080/15548627.2015.1100356 26799652 PMC4835977

[65] Dimauro I, Antonioni A, Mercatelli N, Caporossi D. The role of αB-crystallin in skeletal and cardiac muscle tissues. Cell Stress Chaperones. 2018;23(4):491–505. Epub 2017/11/30. 10.1007/s12192-017-0866-x 29190034 PMC6045558

[66] Fichna JP, Potulska-Chromik A, Miszta P, Redowicz MJ, Kaminska AM, Zekanowski C, et al A novel dominant D109A CRYAB mutation in a family with myofibrillar myopathy affects αB-crystallin structure. BBA Clin. 2017;7:1–7. Epub 2016/11/11. 10.1016/j.bbacli.2016.11.004 27904835 PMC5124346

[67] Markossian KA, Yudin IK, Kurganov BI. Mechanism of suppression of protein aggregation by α-crystallin. Int J Mol Sci. 2009;10(3):1314–45. Epub 2009/03/19. 10.3390/ijms10031314 19399251 PMC2672032

[68] Fujita M, Mitsuhashi H, Isogai S, Nakata T, Kawakami A, Nonaka I, et al Filamin C plays an essential role in the maintenance of the structural integrity of cardiac and skeletal muscles, revealed by the medaka mutant zacro. Dev Biol. 2012;361(1):79–89. Epub 2011/10/14. 10.1016/j.ydbio.2011.10.008 . 22020047

[69] Min JN, Whaley RA, Sharpless NE, Lockyer P, Portbury AL, Patterson C. CHIP deficiency decreases longevity, with accelerated aging phenotypes accompanied by altered protein quality control. Mol Cell Biol. 2008;28(12):4018–25. Epub 2008/04/14. 10.1128/MCB.00296-08 18411298 PMC2423116

[70] Morishima Y, Wang AM, Yu Z, Pratt WB, Osawa Y, Lieberman AP. CHIP deletion reveals functional redundancy of E3 ligases in promoting degradation of both signaling proteins and expanded glutamine proteins. Hum Mol Genet. 2008;17(24):3942–52. Epub 2008/09/10. 10.1093/hmg/ddn296 18784277 PMC2605787

[71] Kley RA, Maerkens A, Leber Y, Theis V, Schreiner A, van der Ven PF, et al A combined laser microdissection and mass spectrometry approach reveals new disease relevant proteins accumulating in aggregates of filaminopathy patients. Mol Cell Proteomics. 2013;12(1):215–27. Epub 2012/10/31. 10.1074/mcp.M112.023176 23115302 PMC3536902

[72] Maerkens A, Kley RA, Olivé M, Theis V, van der Ven PF, Reimann J, et al Differential proteomic analysis of abnormal intramyoplasmic aggregates in desminopathy. J Proteomics. 2013;90:14–27. Epub 2013/04/30. 10.1016/j.jprot.2013.04.026 23639843 PMC5120880

[73] Maerkens A, Olivé M, Schreiner A, Feldkirchner S, Schessl J, Uszkoreit J, et al New insights into the protein aggregation pathology in myotilinopathy by combined proteomic and immunolocalization analyses. Acta Neuropathol Commun. 2016;4:8 Epub 2016/02/03. 10.1186/s40478-016-0280-0 26842778 PMC4739336

[74] Olivé M, Kley RA, Goldfarb LG. Myofibrillar myopathies: new developments. Curr Opin Neurol. 2013;26(5):527–35. 10.1097/WCO.0b013e328364d6b1 23995273 PMC5127196

[75] Béhin A, Salort-Campana E, Wahbi K, Richard P, Carlier RY, Carlier P, et al Myofibrillar myopathies: State of the art, present and future challenges. Rev Neurol (Paris). 2015;171(10):715–29. Epub 2015/09/03. 10.1016/j.neurol.2015.06.002 . 26342832

[76] Winter L, Goldmann WH. Biomechanical characterization of myofibrillar myopathies. Cell Biol Int. 2015;39(4):361–3. Epub 2014/12/03. 10.1002/cbin.10384 . 25264173

[77] Kley RA, Olivé M, Schröder R. New aspects of myofibrillar myopathies. Curr Opin Neurol. 2016;29(5):628–34. 10.1097/WCO.0000000000000357 . 27389816

[78] Batonnet-Pichon S, Behin A, Cabet E, Delort F, Vicart P, Lilienbaum A. Myofibrillar Myopathies: New Perspectives from Animal Models to Potential Therapeutic Approaches. J Neuromuscul Dis. 2017;4(1):1–15. 10.3233/JND-160203 28269794 PMC5345645

[79] Fichna JP, Maruszak A, Żekanowski C. Myofibrillar myopathy in the genomic context. J Appl Genet. 2018;59(4):431–9. Epub 2018/09/10. 10.1007/s13353-018-0463-4 . 30203143

[80] Schröder R. Protein aggregate myopathies: the many faces of an expanding disease group. Acta Neuropathol. 2013;125(1):1–2. 10.1007/s00401-012-1071-8 . 23224320

[81] Carra S, Boncoraglio A, Kanon B, Brunsting JF, Minoia M, Rana A, et al Identification of the Drosophila ortholog of HSPB8: implication of HSPB8 loss of function in protein folding diseases. J Biol Chem. 2010;285(48):37811–22. Epub 2010/09/21. 10.1074/jbc.M110.127498 20858900 PMC2988385

[82] Green N, Walker J, Bontrager A, Zych M, Geisbrecht ER. A tissue communication network coordinating innate immune response during muscle stress. J Cell Sci. 2018;131(24). Epub 2018/12/18. 10.1242/jcs.217943 30478194 PMC6307882

[83] Geisbrecht ER, Haralalka S, Swanson SK, Florens L, Washburn MP, Abmayr SM. Drosophila ELMO/CED-12 interacts with Myoblast city to direct myoblast fusion and ommatidial organization. Dev Biol. 2008;314(1):137–49. 10.1016/j.ydbio.2007.11.022 18163987 PMC2697615

[84] Sokol NS, Cooley L. Drosophila filamin encoded by the cheerio locus is a component of ovarian ring canals. Curr Biol. 1999;9(21):1221–30. 10.1016/s0960-9822(99)80502-8 . 10556087

[85] Friedrich MV, Schneider M, Timpl R, Baumgartner S. Perlecan domain V of Drosophila melanogaster. Sequence, recombinant analysis and tissue expression. Eur J Biochem. 2000;267(11):3149–59. 10.1046/j.1432-1327.2000.01337.x . 10824099

[86] Wang ZH, Clark C, Geisbrecht ER. Drosophila Clueless is involved in Parkin-dependent mitophagy by promoting VCP-mediated Marf degradation. Hum Mol Genet. 2016 10.1093/hmg/ddw067 . 26931463 PMC5062585

[87] Brooks DS, Vishal K, Kawakami J, Bouyain S, Geisbrecht ER. Optimization of wrMTrck to monitor Drosophila larval locomotor activity. J Insect Physiol. 2016;93–94:11–7. Epub 2016/07/16. 10.1016/j.jinsphys.2016.07.007 . 27430166 PMC5722213

