# Supplementary material for: Drosophila NUAK functions with Starvin/BAG3 in autophagic protein turnover
Source: PLoS Genet. 2020 Apr 22;16(4):e1008700. doi: 10.1371/journal.pgen.1008700 (PMC7176095; doi:10.1371/journal.pgen.1008700)
Supplement: S1 Table — Graph type, n values, statistical tests and p-values for all quantitative analysis. (DOCX) [file pgen.1008700.s001.docx]

**Table S1. Raw data and statistics summary**

| **Panel** | **Graph type** | **N value** | **Statistical test used** | **Precision** | **p-value** |
| --- | --- | --- | --- | --- | --- |
| Fig. 1E | Box and whisker plot | N≥19 for each genotype | One-way ANOVA  Kruskal-Wallis test | Min to max | p<0.001 |
| Fig. 1F | Scatter plot | N≥21 for each genotype | One-way ANOVA  Kruskal-Wallis test | Mean +/- SD | p<0.001 |
| Fig. 2G | Scatter plot | N≥24 for each genotype | One-way ANOVA  Kruskal-Wallis test | Mean +/- SD | p<0.001 |
| Fig. 2H | Scatter plot | N=20 for each genotype | Unpaired t-test | Mean +/- SD | p<0.001 |
| Fig. 2K | Scatter plot | N≥35 for each genotype | Unpaired t-test | Mean +/- SD | n.s. |
| Fig. 2L | Scatter plot | N≥38 for each genotype | Unpaired t-test | Mean +/- SD | p<0.001 |
| Fig. 3A | Column bar graph | N=22 for each genotype | Unpaired t-test | Mean +/- SEM | p<0.0001 |
| Fig. 4K | Box and whisker plot | N≥20 for each genotype | One-way ANOVA  Kruskal-Wallis test | Min to max | p<0.05, p<0.01, p<0.001 |
| Fig. 6F | Scatter plot with bar | N≥19 for each genotype | One-way ANOVA  Kruskal-Wallis test | Mean +/- SD | p<0.005, p<0.001 |
| Fig. 6I | Box and whisker plot | N≥21 for each genotype | One-way ANOVA  Kruskal-Wallis test | Min to max | p<0.001 |
| Fig. 6N | Column bar graph | N≥21 for each genotype | One-way ANOVA  Kruskal-Wallis test | Mean +/- SD | p<0.005, p<0.001 |
| Fig. 7B | Scatter plot with bar | N≥12 for each genotype | One-way ANOVA  Kruskal-Wallis test | Mean +/- SD | p<0.001, p<0.001 |
| Fig. 7E | Box and whisker plot | N≥19 for each genotype | One-way ANOVA  Kruskal-Wallis test | Mean +/- SD | p<0.001 |
| Fig. 8D | Scatter plot | N≥17 for each genotype | One-way ANOVA  Kruskal-Wallis test | Mean +/- SD | p<0.001 |
| Fig. 8H | Scatter plot | N≥25 for each genotype | One-way ANOVA  Kruskal-Wallis test | Mean +/- SD | p<0.001 |
| Fig. 8I | Column bar graph | Pool of 3 larvae per genotype subjected to each condition (N=3 biological replicates and N=3 technical replicates) | N/A | Mean +/- SEM | N/A |
| Fig. 10A | Column bar graph | Pool of 3 larvae per genotype subjected to each condition (N=3 biological replicates and N=3 technical replicates) | N/A | Mean +/- SEM | N/A |
| Fig. 11A | Box and whisker plot | N≥20 for each genotype | One-way ANOVA  Kruskal-Wallis test | Mean +/- SD | p<0.001 |
| Fig. 11B | Grouped bar graph | N≥20 for each genotype | Unpaired Mann-Whitney t-test | Mean +/- SEM | p<0.01 |
| **Panel** | **Graph type** | **N value** | **Statistical test used** | **Precision** | **p-value** |
| Fig. S2C | Column bar graph | Pool of 3 larvae per genotype subjected to each condition (N=3 biological replicates and N=3 technical replicates) | Unpaired Mann-Whitney t-test | Mean +/- SD | p<0.01, p<0.005 |
| Fig. S2D | Box and whisker plot | N≥20 for each genotype | One-way ANOVA  Kruskal-Wallis test | Mean +/- SD | p<0.001 |
| Fig. S3E | Scatter plot | N≥20 for each genotype | One-way ANOVA  Kruskal-Wallis test | Mean +/- SD | p<0.001 |
| Fig. S3F | Scatter plot | N≥22 for each genotype | One-way ANOVA  Kruskal-Wallis test | Mean +/- SD | p<0.001 |
| Fig. S5C | Column bar graph | Pool of 3 larvae per genotype subjected to each condition (N=3 biological replicates and N=3 technical replicates) | Unpaired Mann-Whitney t-test | Mean +/- SD | p<0.01 |
| Fig. S6C | Column bar graph | N≥17 for each genotype | One-way ANOVA  Kruskal-Wallis test | Mean +/- SD | P<0.05, p<0.001 |
| Fig. 6D | Scatter plot | N≥20 for each genotype | One-way ANOVA  Kruskal-Wallis test | Mean +/- SD | n.s. |
| Fig. 6E | Scatter plot | N≥20 for each genotype | One-way ANOVA  Kruskal-Wallis test | Mean +/- SD | n.s. |
| Fig. S7B | Column bar graph | Pool of 3 larvae per genotype subjected to each condition (N=3 biological replicates and N=3 technical replicates) | Unpaired Mann-Whitney t-test | Mean +/- SD | p<0.05 |
| Fig. S7D | Column bar graph | Pool of 3 larvae per genotype subjected to each condition (N=3 biological replicates and N=3 technical replicates) | Unpaired Mann-Whitney t-test | Mean +/- SD | p<0.01 |
| Fig. S8A | Column bar graph | Pool of 3 larvae per genotype subjected to each condition (N=3 biological replicates and N=3 technical replicates) | Unpaired Mann-Whitney t-test | Mean +/- SD | p<0.01 |
