# Supplementary figures and images for: Drosophila NUAK functions with Starvin/BAG3 in autophagic protein turnover
Source: PLoS Genet. 2020 Apr 22;16(4):e1008700. doi: 10.1371/journal.pgen.1008700 (PMC7176095; doi:10.1371/journal.pgen.1008700)

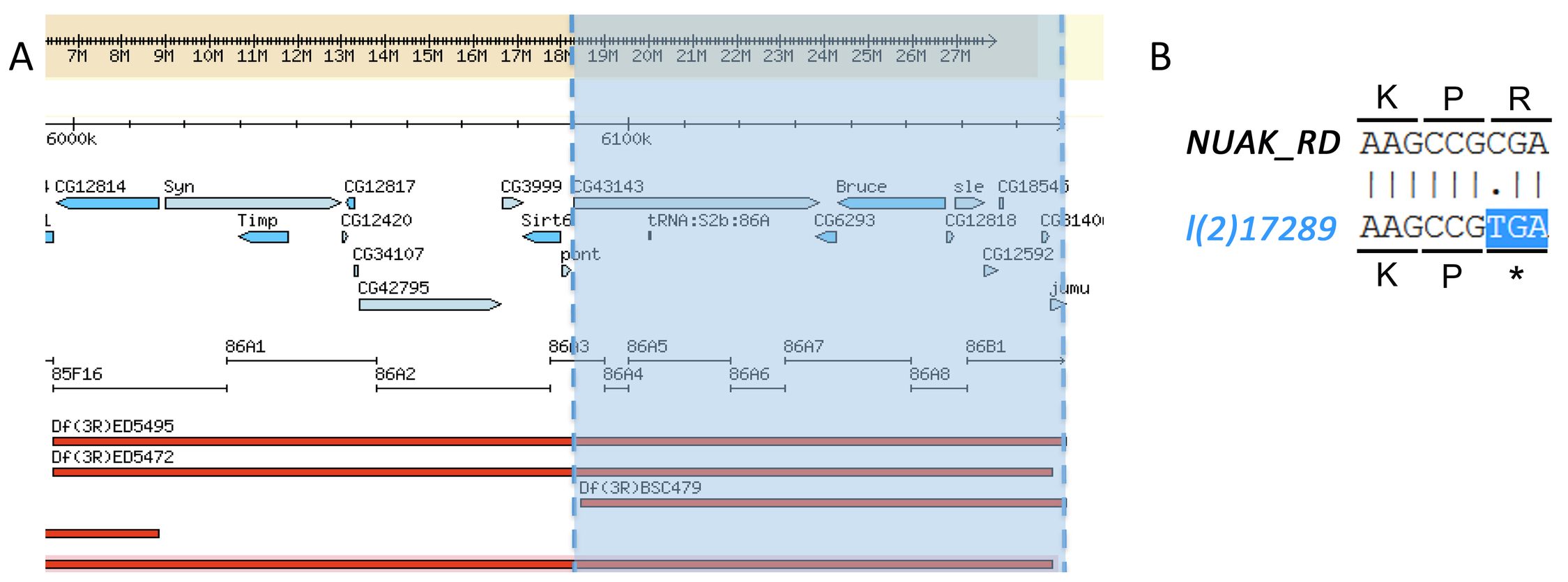

Supplement: S1 Fig — (A) Deficiency mapping narrowed down the l(3) 17289 mutation to Df(3R)BSC479. This deficiency removes ten genes. (B) RT-PCR and Sanger sequencing of CG43143 revealed a C>T change that results in a stop codon. (TIF) [file pgen.1008700.s002.tif]

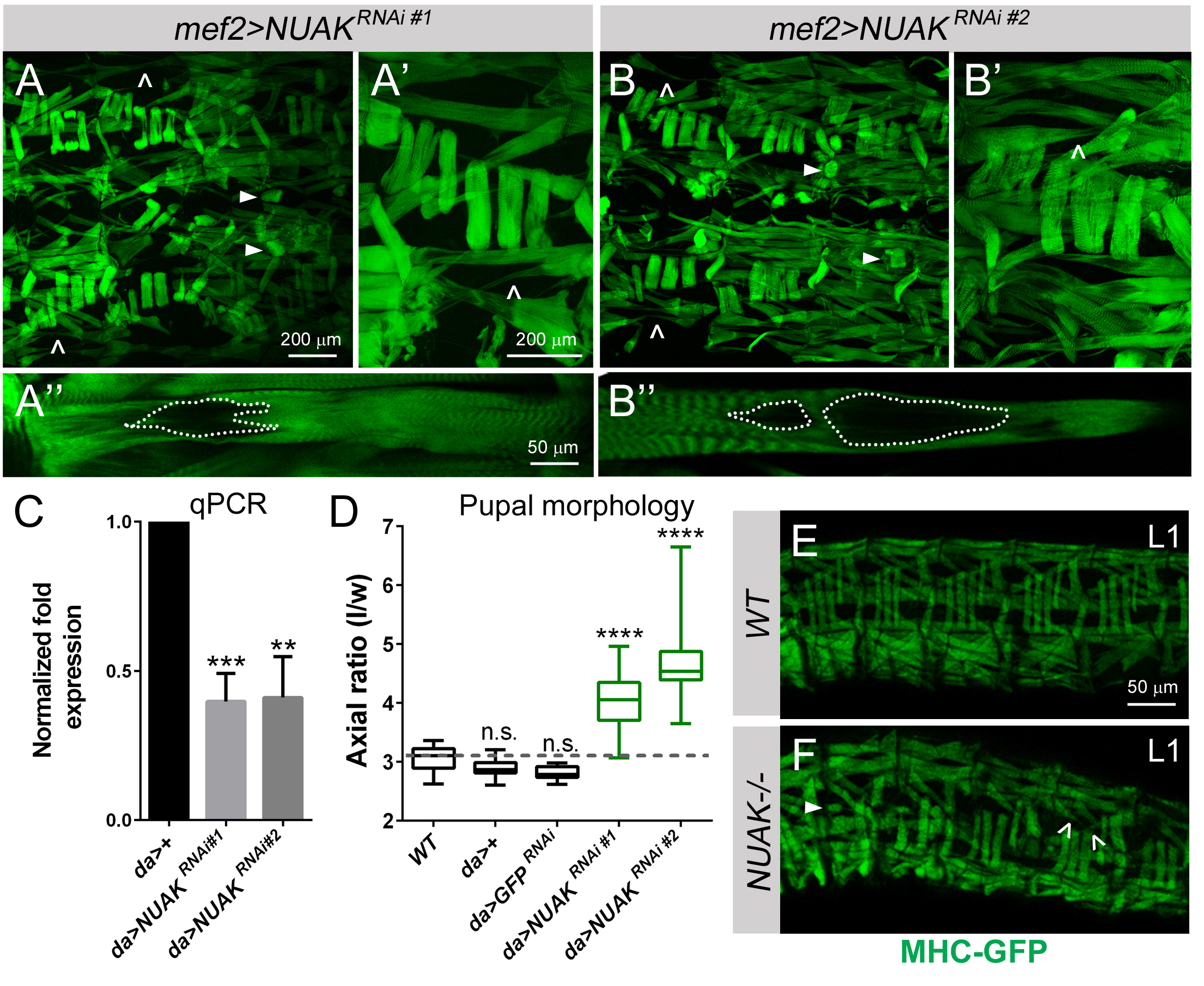

Supplement: S2 Fig — (A-B’) L3 muscle fillets stained with phalloidin after knockdown of NUAK using two independent UAS-RNAi insertions (UAS-NUAK RNAi #1 or UAS-NUAK RNAi #2). (A-B’) 4x magnification (A,B) or 10x view of one hemisegment (A’,B’) shows thinner (carets) or detached (arrowhead) muscles. (A”,B”) 20x image of a representative VL3 muscle show areas lacking F-actin (white dotted outline). (C) qPCR verifies that NUAK transcript levels are reduced ~50% after induction of either UAS-NUAK RNAi #1 or UAS-NUAK RNAi #2 using the ubiquitous daughterless (da)-Gal4 driver. (D) Box and whisker plot depicting axial ratios of pupal length upon ubiquitous knockdown of NUAK RNAi with da-Gal4. (E,F) Whole mount L1 larval muscles visualized with MHC-GFP. Thinner (white carets) or altered muscle pattern (white arrowhead) is observed upon loss of NUAK. Mean +/- SEM (**, p<0.01; ***, p<0.005; ****, p<0.001). (TIF) [file pgen.1008700.s003.tif]

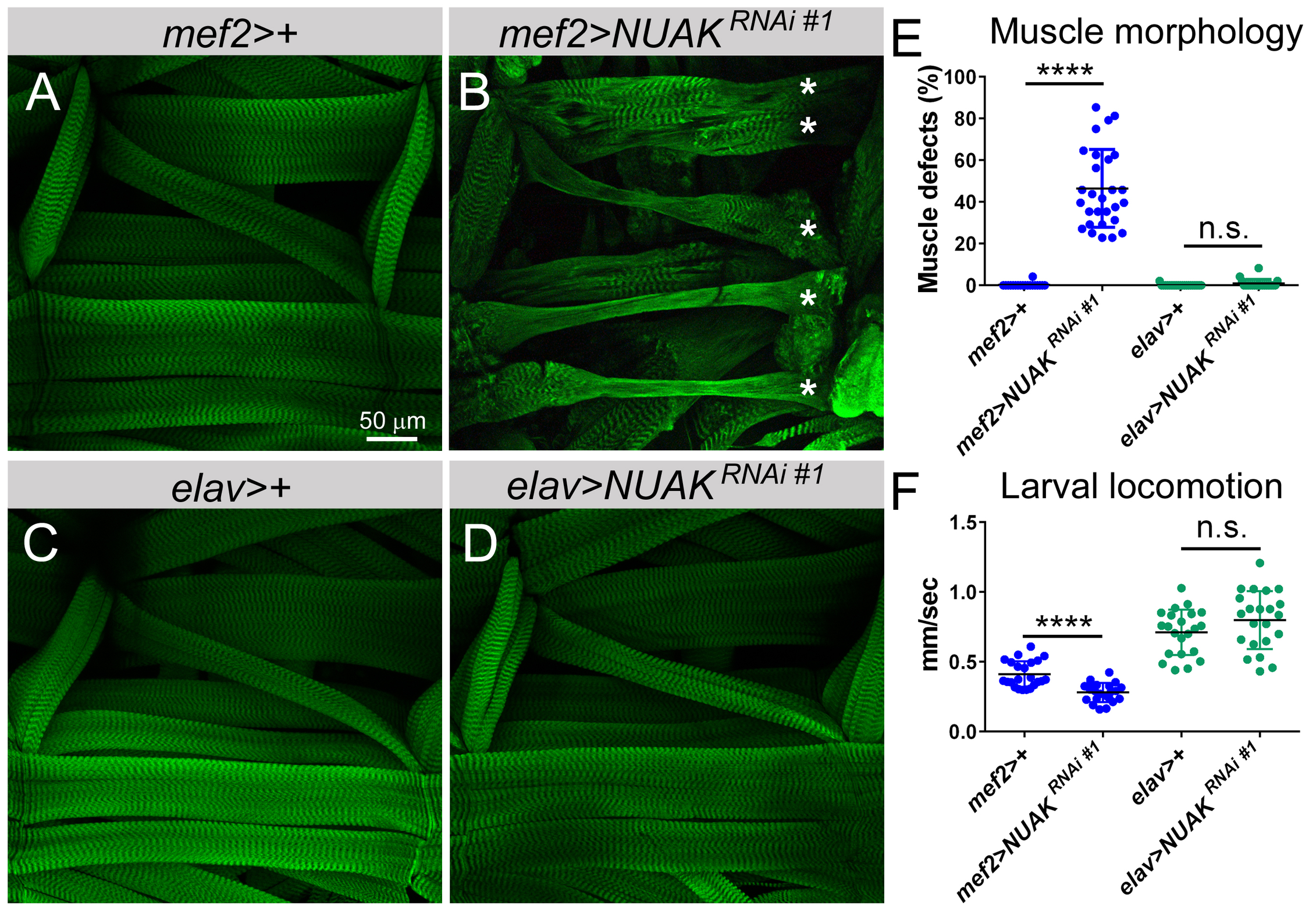

Supplement: S3 Fig — (A-D) L3 muscles within a single hemisegment stained with phalloidin. (A) Expression of the mef2 driver alone shows no phenotype. (B) mef2-driven NUAK RNAi results in morphological muscle defects. (C,D) Neither the neuronal driver C155 alone (C) or inducing NUAK RNAi (D) causes muscle phenotypes. (E) Scatter plot shows that muscle defects are only apparent upon NUAK RNAi induction in muscle, but not neuronal tissue. (F) Scatter plot of larval locomotor ability upon muscle (mef2) or neuronal (C155) RNAi knockdown of NUAK. Larvae were transferred from 25°C to 29°C after hatching. Mean +/- SEM (*, p<0.05; ****, p<0.001; n.s., not significant). (TIF) [file pgen.1008700.s004.tif]

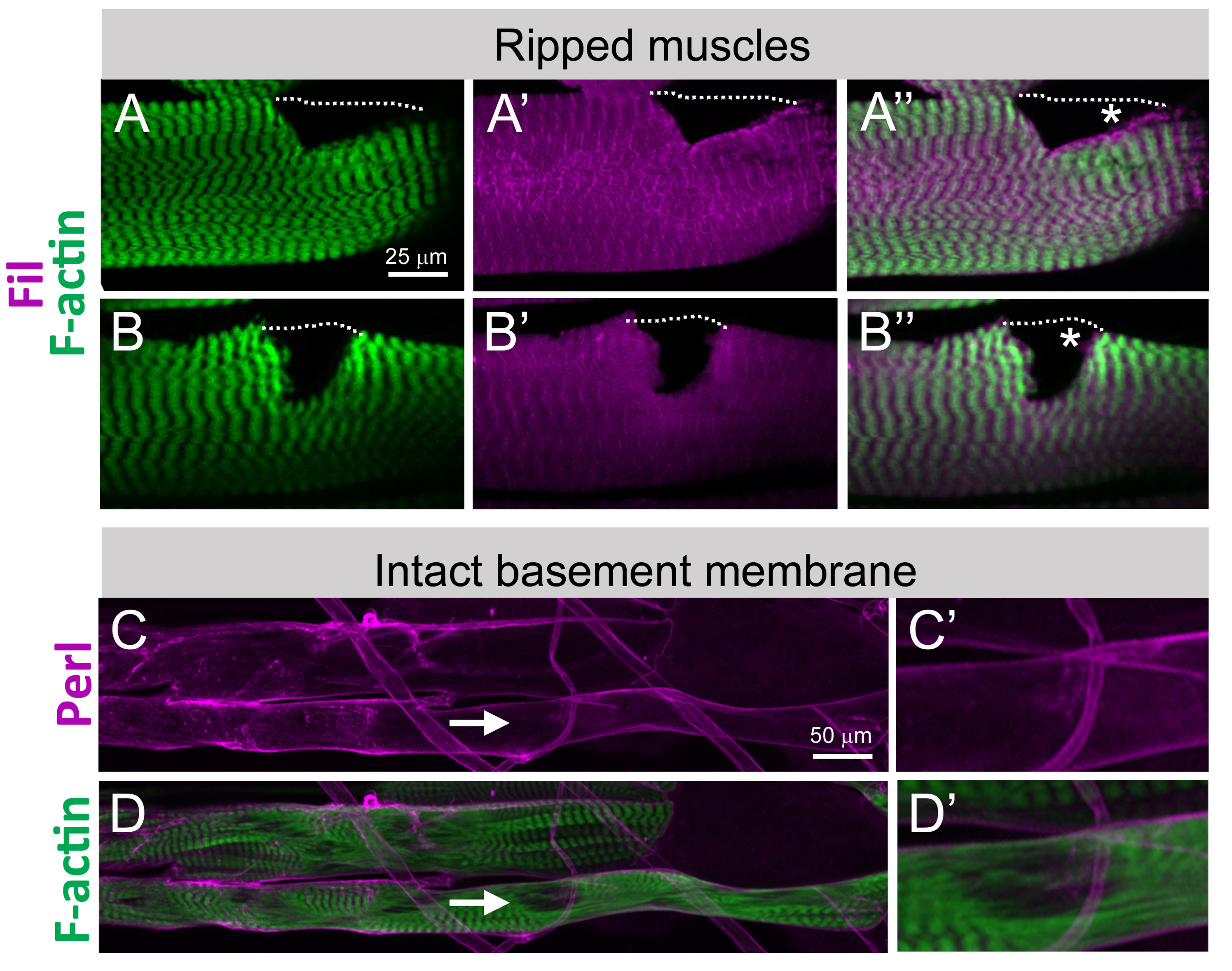

Supplement: S4 Fig — (A-B”) Intentionally torn L3 muscles stained for F-actin (green) do not accumulate Fil protein (purple, asterisk). (C,D’) NUAK-/- with regions devoid of F-actin (green, white arrow) have an intact basement membrane visualized by Perlecan (purple). (TIF) [file pgen.1008700.s005.tif]

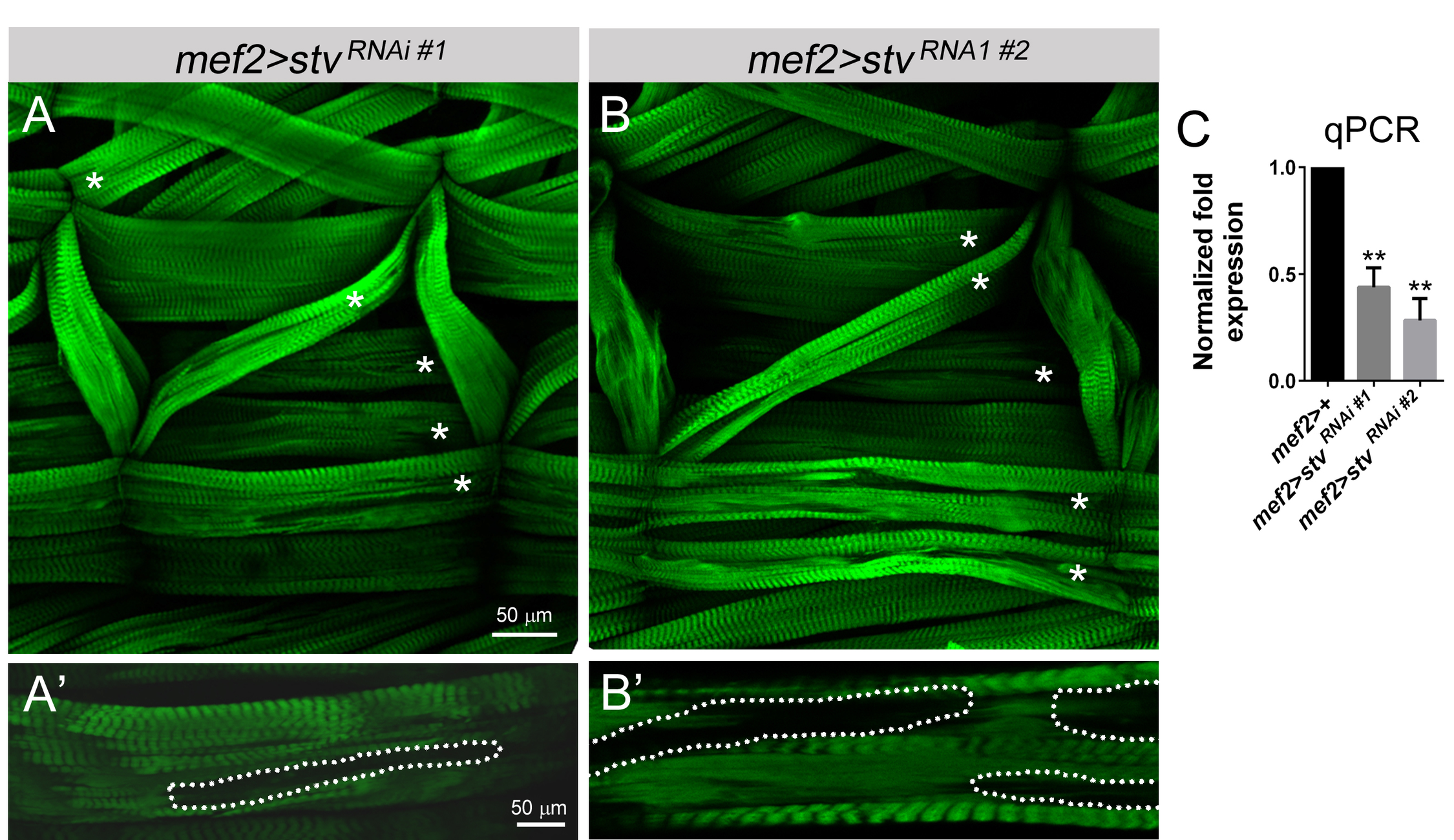

Supplement: S5 Fig — (A-B’) 10x or 20 x images of a single hemisegment of the L3 musculature stained with phalloidin (green). Expression of both stv RNAi insertions in muscle show regions that where F-actin is excluded (* in A,B; white dotted lines in A’,B’). (C) Both the UAS-stv RNAi #1 or UAS-stvRNAi #2 RNAi lines effectively decrease stv mRNA levels as assayed by qPCR. Mean +/- SEM (**, p<0.01). (TIF) [file pgen.1008700.s006.tif]

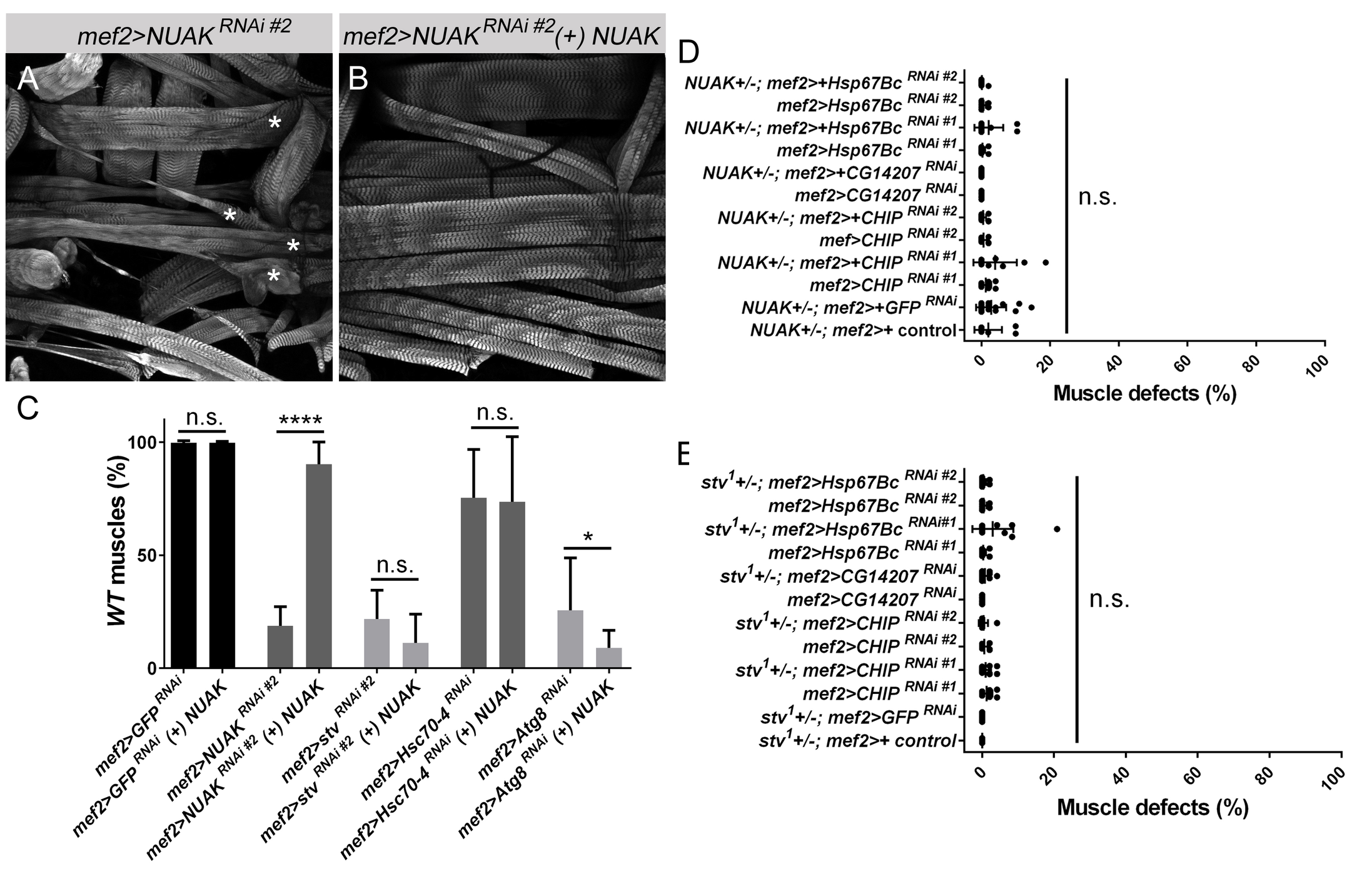

Supplement: S6 Fig — (A,B) One hemisegment of the L3 musculature stained with phalloidin. Defects caused by knockdown of NUAK RNAi (A) can be rescued upon re-introduction of NUAK cDNA in muscle tissue. (C) Bar graph showing NUAK rescue results. NUAK is capable of restoring muscle defects due to loss of NUAK, but not Stv, Hsc70-4, or Atg8a. (D,E) Scatter plots of genetic interactions with NUAK (D) or stv (E). Mean +/- SEM (*, p<0.05; ****, p<0.001). (TIF) [file pgen.1008700.s007.tif]

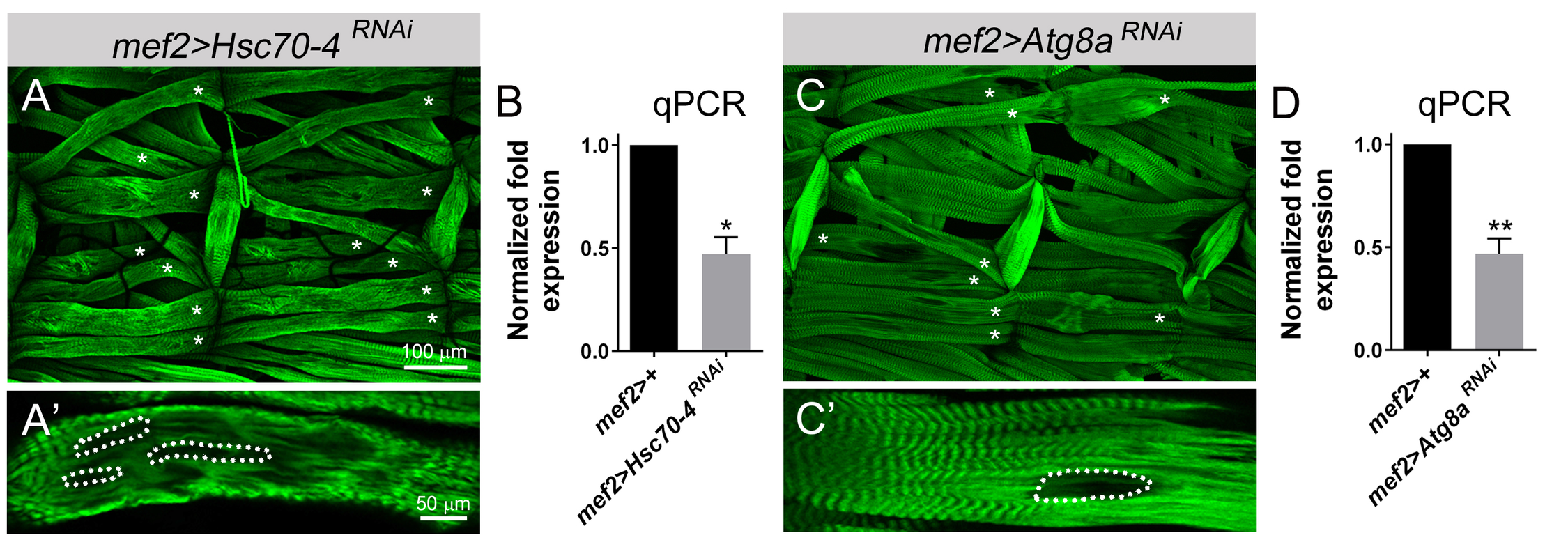

Supplement: S7 Fig — (A,C) F-actin labeled muscles in two hemisegments of the L3 musculature. (A) Nearly all muscles of the genotype mef2>Hsc70-4 show abnormal morphology (*). (A’) Regions devoid of F-actin are outlined (white dashed lines). (B) Bar graph shows a decrease in Hsc70-4 mRNA levels driven with mef2-Gal4. (C) RNAi knockdown of Atg8a mRNA affects muscles to a lesser extent. (C’) The predominant phenotype is the presence of dark regions, indicative of protein aggregation. (D) Bar graph illustrating that the UAS-Atg8a RNAi insertion effectively reduces transcript levels. Mean +/- SEM (*, p<0.05; **, p<0.01). (TIF) [file pgen.1008700.s008.tif]

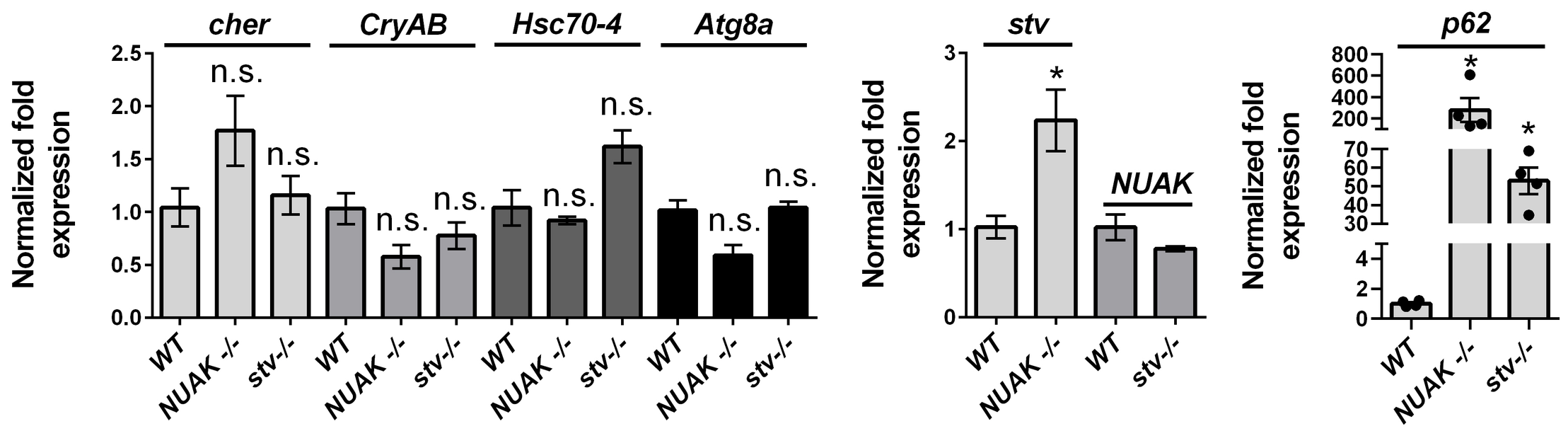

Supplement: S8 Fig — Bar graphs showing the indicated transcripts in NUAK-/- or stv-/-. cher, CryAB, Hsc70-4, and Atg8a mRNA levels are not altered upon loss of NUAK or Stv (left panel). stv transcript levels are mildly increased in NUAK mutants, but NUAK transcripts do not change upon loss of Stv (middle panel). p62 mRNA levels are much higher in both NUAK and stv mutants (right panel). Mean +/- SEM (*, p<0.05; **, p<0.01; n.s., not significant). (TIF) [file pgen.1008700.s009.tif]

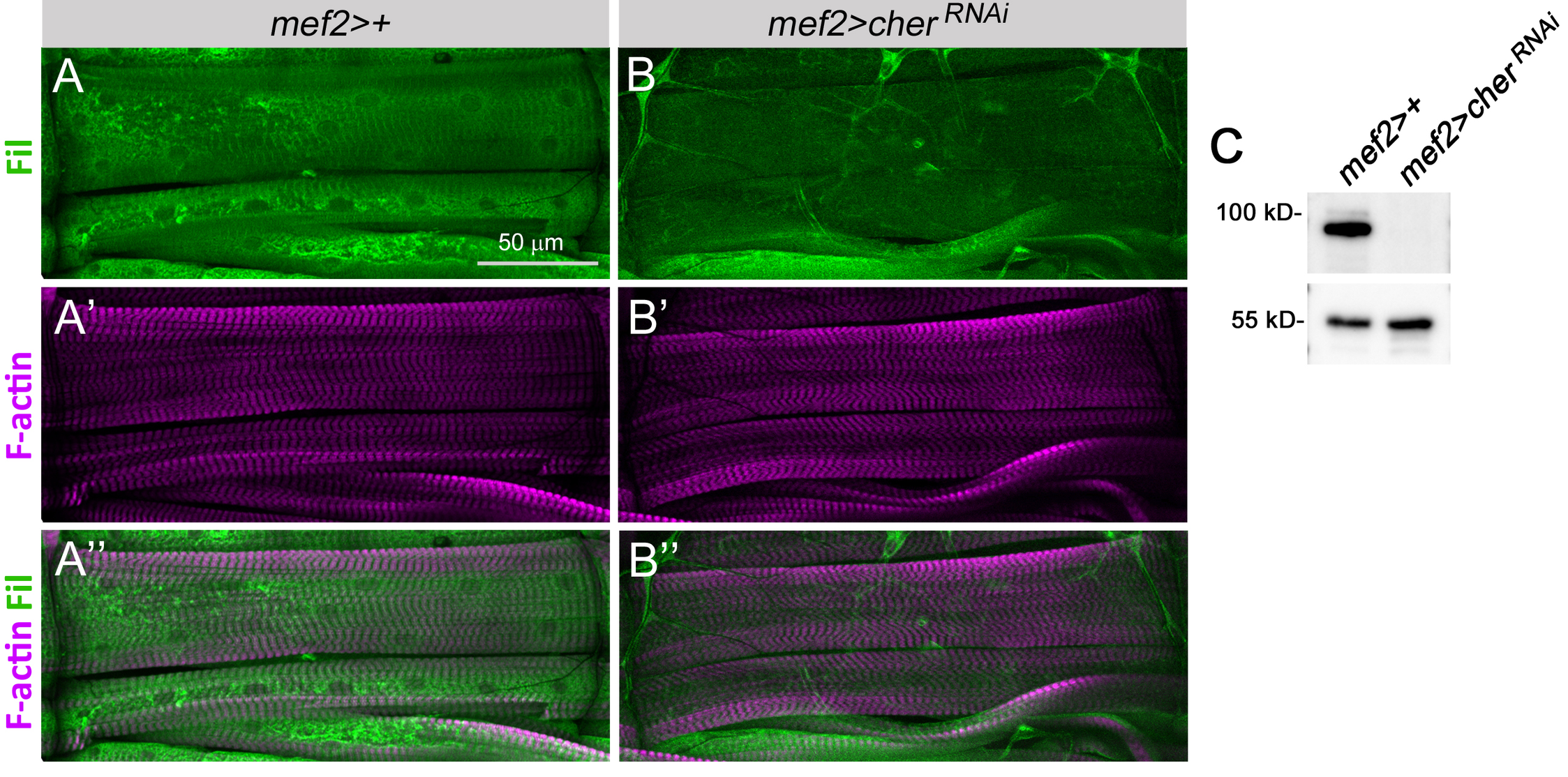

Supplement: S9 Fig — (A-B”) Anti-Fil (green) and F-actin (purple) staining of L3 muscles VL3 and VL4 in control (mef2>+) or upon a decrease in cher mRNA levels (mef2>cherRNAi). The striated pattern of Fil immunostaining (A,A”) is blunted upon targeted induction of cher RNAi in muscle tissue (B, B”). (C) Western blot showing a decrease in the 90 kD form of Fil after knockdown of cher transcripts. (TIF) [file pgen.1008700.s010.tif]
